# Reconsidering the Enzyme Kinetics of [FeFe]-Hydrogenases: Improved Turnover Rates and New Insights into pH and Potential Dependence with Eu(II)-Based Solution Assays

**DOI:** 10.1021/acs.analchem.5c02898

**Published:** 2025-11-25

**Authors:** Eda Sönmez, Nikolaos Kostopoulos, Mira Gamache, Mun Hon Cheah, Ping Huang, Andrew J. Bagnall, Dawit T. Filmon, Ivan Voloshyn, Thomas Happe, Moritz Senger, Nicolas Plumeré, Alina Sekretareva, Gustav Berggren

**Affiliations:** † Department of Chemistry—Ångström, Molecular Biomimetics, 8097 Uppsala University, Box 523, 75120 Uppsala, Sweden; ‡ TUM Campus Straubing for Biotechnology and Sustainability - 9184 Technical University of Munich, Uferstrasse 53, 94315 Straubing, Germany; § Department of Plant Biochemistry, Faculty of Biology and Biotechnology, Photobiotechnology, 9142 Ruhr University Bochum, Universitätsstrasse 150, 44801 Bochum, Germany; ∥ Department of Chemistry—BMC, Biochemistry, Uppsala University, Box 523, 75120 Uppsala, Sweden

## Abstract

Metal-dependent redox enzymes are central for microbial processing of gases, as exemplified by hydrogenase, nitrogenase, and carbon monoxide dehydrogenase. Due to their remarkable efficiencies and high biotechnological relevance, such gas-processing enzymes are intensively studied. Nevertheless, many of their mechanistic details remain opaque. We herein report a new method for solution assays under reducing conditions based on europium­(II) as a terminal reductant and show how it can be employed to gain new insight into hydrogenase kinetics. Compared with the commonly used reductant sodium dithionite, this work shows that Eu­(II) can serve as a robust and relatively easy-to-handle alternative electron donor, also providing a larger potential window for catalytic studies. Further, this work clarifies previous discrepancies in the literature regarding the influence of pH on hydrogenase kinetics in these assays. Our study shows that sodium dithionite, most likely due to its decomposition into SO_2_, alters hydrogenase kinetics in solution assays. Using [FeFe]-hydrogenase I from *Clostridium pasteurianum* (CpI) as a model system, Eu­(II)-based solution assays demonstrated a pH optimum of 5–6 and rates greatly exceeding those observed with sodium dithionite assays. The higher turnover frequencies observed at low pH obtained with Eu­(II) align more closely with the electrochemical data. Additionally, a strong driving force dependency was identified. A solution potential change of approximately 180 mV resulted in a 35-fold increase in the catalytic rate, yielding activities far surpassing those of earlier reports on CpI turnover frequencies. These findings provide new insight into the pH dependence and overall kinetic performance of [FeFe]-hydrogenases. More broadly, the report outlines alternative assay methods employing Eu­(II) to better understand the enzyme kinetics of hydrogenases and related metalloenzymes.

## Graphical abstract

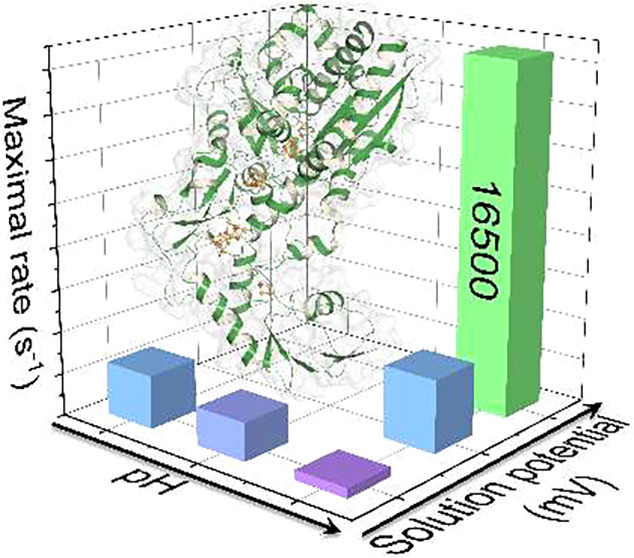

## Introduction

Metalloenzymes involved in the processing of N_2_, CO_2_, and H_2_ are intensively studied due to their unique cofactors and potential biotechnological relevance. Hydrogenases provide a striking example of this.^4,5^ These enzymes are central to microbial hydrogen metabolism, where they catalyze the reversible interconversion of H^+^/H_2_, providing a possible route for (photo)­biological H_2_ production but also enabling the use of H_2_ as an electron supply for a range of CO_2_ reducing metabolic pathways.^6^ The [FeFe]-hydrogenases are found in all domains of life and are commonly associated with fermentative H_2_ gas production.^7^ Most of the to-date characterized [FeFe]-hydrogenases are fast and reported to catalyze both proton reduction and H_2_ oxidation with minimal energy losses.^8,9^ Their reactivity is enabled by a biologically unique hexanuclear iron-cofactor, called the H-cluster (Figure 1).^10–12^ [FeFe]-hydrogenases commonly also contain additional [4Fe-4S] and/or [2Fe-2S] clusters serving as electrical wires connecting the deeply buried active site to the protein surface.^13,14^ Due to their high activity, [FeFe]-hydrogenases have been of high interest within sustainable H_2_ production research both as biocatalysts in photobiological and biohybrid systems and as templates for catalytic mimics.^15–17^

**Figure 1:**
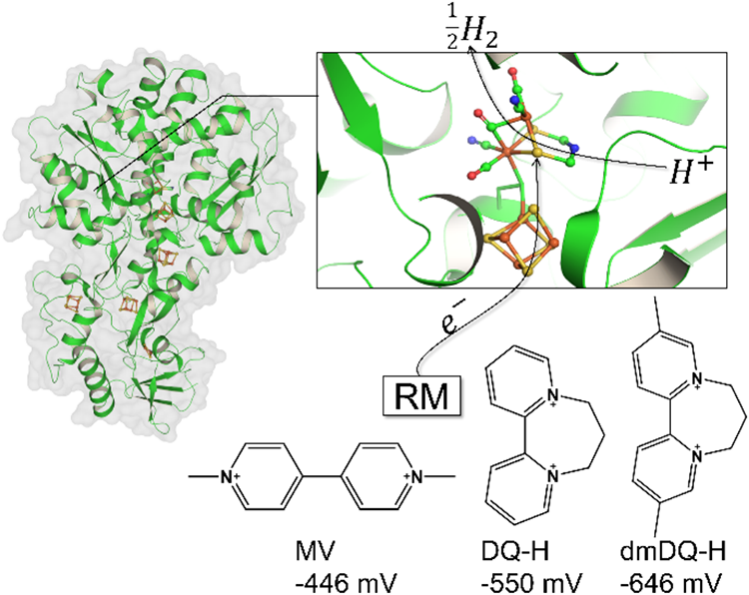
Visualization of [FeFe]-hydrogenase I from *Clostridium pasteurianum* with a detailed view of the H-cluster on top right (PDB: 6GM2).^1^ Color coding: Fe: orange, S: yellow, O: red, N: blue, and C: green. Bottom right shows the redox mediators (RM) used in this study and the respective reported formal reduction potentials (*E*°′) vs SHE for their RM^2+^/RM^•+^ redox couple.^2,3^

In order to unlock their full potential, a solid mechanistic understanding of their reactivity is necessary, and detailed kinetic information is certainly a key aspect of this work. As redox enzymes, they are highly amenable to studies using electrochemistry, and in particular, protein film electrochemistry (PFE) has provided extensive information on the details of, e.g., inhibition, catalytic bias, and overpotential requirements of these enzymes.^18,19^ However, it is extremely challenging to determine actual turnover frequencies (TOFs) using PFE; while observed current directly correlates with enzymatic rates, the amount of enzyme on the electrode surface is generally unknown. Moreover, there are limited data on the effect of surface immobilization on overall enzyme properties. Solution assays can thus provide complementary enzyme kinetic information, most importantly, TOFs, and avoid potential issues associated with immobilization. Despite their obvious importance, detailed solution assays probing both pH and driving force dependency of [FeFe]-hydrogenases are rare. To our knowledge, only one study by van Dijk et al. extensively studied both of these dependencies.^20^ They reported an increase in H_2_ production rates with lower pH by the [FeFe]-hydrogenase from *Megasphaera elsdenii*, as well as an S-shaped solution potential dependence. More specifically, rates were reported to show *dependence* on solution potential only in a rather limited potential window, and a rate *independent* of solution potential was already observed when potentials were increased 45–50 mV beyond the onset potential for catalysis. However, the activities were determined using single-concentration data point analysis with regard to the redox mediator and consequently do not necessarily reflect the true *V*_max_ of the enzyme under the specific assay conditions.

Furthermore, when reported, solution assays generally employ sodium dithionite (Na_2_S_2_O_4_, annotated NaDT) as the terminal electron donor (see, e.g., refs 1,20–22 ). Albeit a convenient reductant, its use in this context is problematic. The reduction potential of NaDT is often cited as −0.66 V vs SHE, but it is known to vary with both pH and NaDT concentration as the actual electron donor is the SO_2_^•–^ species existing in equilibrium with S_2_O_4_^2–^.^23–25^ The complexity of NaDT is further compounded by the fact that its effect on biomolecules has been far from fully elucidated. When NaDT is oxidized under anaerobic aqueous conditions, a variety of sulfur oxide species are formed (SO_3_^2–^, SO_4_^2–^, SO_2_, etc.), which could have an effect on biomolecules and their mechanisms.^26^ The unwanted effect(s) of these byproducts on enzymes such as DMSO reductase,^27^ nitrite reductase,^28^ and acetyl-coenzyme A^29^ have been reported previously. Similarly, the presence of NaDT and its oxidation products has been shown to interfere with kinetic studies of nitrogenase.^23^ Specifically for hydrogenase, Martini et al. recently showed using spectroscopic and electrochemical methods that NaDT appeared to have an inhibitory effect on [FeFe]-hydrogenase.^24^ The observation by Martini et al. that the inhibition increased at lower pH strongly suggests that the actual inhibitor is SO_2_, originating from SO_3_^2–^, an oxidation product of NaDT. Potential hazards with employing NaDT in hydrogenase assays are further implied upon comparison of kinetic data collected using electrochemistry and solution assays. Indeed, in a rare example, electrochemically measured turnover frequencies of up to 21000 ± 12000 s^–1^ have been reported for the [FeFe]-hydrogenase from *Clostridium acetobutylicum* (*Ca*HydA).^30^ This was achieved through a combination of electrochemical scanning tunneling microscopy (EC-STM) techniques and macroscopic electrochemical measurements, and the estimated TOF is often cited as the maximum rate reported for these enzymes. However, solution assays of *Ca*HydA give a 10-fold lower TOF of ∼2000 s^–1^,^31^ while an almost 20-fold lower TOF of 1200 s^–1^ is often cited for the highly homologous hydrogenase I from *C. pasteurianum* (CpI).^32^ In both the latter examples, NaDT is employed as terminal electron donor in combination with methyl viologen (MV) as the redox mediator. Related to this, the apparent catalytic bias of [FeFe]-hydrogenase, i.e., the relative rates of H_2_ oxidation and formation under conditions of high driving force, often differs between solution assays and PFE data. Lastly, PFE experiments generally show lower pH optima for H_2_ gas production relative to solution assays.^33^

Improved solution assays are necessary to clarify whether these differences in reported rates and pH dependence are due to alteration of the enzyme’s properties arising from surface immobilization or due to suboptimal solution assay conditions. In an effort to address this issue, we have systematically explored Eu­(II) as an alternative electron donor. We show that Eu­(II) complexed with EGTA (3,12-bis­(carboxymethyl)-6,9-dioxa-3,12-diazatetradecane-1,14-dioic acid) provides numerous advantages compared with NaDT. Eu­(II)­EGTA has previously been reported in related contexts, e.g., as a terminal reductant for FeS clusters^34^ and as a reductant in catalytic assays with hydrogenase mimics.^35^ Here, we expand on these reports and study their suitability with regard to pH range and compatibility with three different redox mediators (RMs) with varying reduction potential. In short, compared with other reductants, Eu­(II)­EGTA is relatively easy to prepare and stable on a time scale of hours in solution. When combined with suitable redox mediators, it enables assays in a pH range appropriate for many enzymes and provides a wider window for studying the solution potential influence on catalytic rates compared with NaDT.

Furthermore, using the CpI hydrogenase as a model system, we demonstrate how Eu­(II)­EGTA-based assays reveal a clearly distinct pH dependence relative to assays employing NaDT. We also show that both NaDT and SO_3_^2–^ act as inhibitors in solution assays with [FeFe]-hydrogenase at pH < 8, most likely via formation of SO_2_, rationalizing the difference observed between the two terminal reductants. Moreover, the low reduction potential of Eu­(III/II)­EGTA (−0.88 V vs SHE)^34^ enables the use of diquat-based redox mediators with reduction potentials more negative than the commonly employed MV^2+^, which allows the exploration of a larger solution potential window than previously reported for [FeFe]-hydrogenase. The three employed redox mediators, MV^2+^, the unsubstituted diquat (DQ-H^2+^), and the dimeta-methyl-substituted diquat (dmDQ-H^2+^), are shown in Figure 1, together with their reported E°′ (the published formal reduction potential of dmDQ-H^2+^/dmDQ-H^•+^ varies by 22 mV; therefore, the average −0.646 V ± 0.016 V was used).^2,3^ Our results show that the pH dependence of CpI in Eu­(II)-based solution assays correlates well with the PFE data. Moreover, the rate was found to be strongly dependent on the solution potential, resulting in peak activities which were more than 10-fold higher than those previously reported. In combination, these findings pave the way for improved kinetic modeling and new insights into the mechanism of [FeFe]-hydrogenase. More broadly, the study underscores the importance of shifting away from NaDT as an electron donor in enzyme assays and provides a protocol for achieving this.

## Experimental Section

### General

All chemicals were purchased from Sigma-Aldrich or VWR, unless otherwise stated. Specifically, sodium dithionite (≥82%), EuCl_2_ (99.99%), and EGTA (≥98%) were all purchased from Sigma-Aldrich, with reported purities given in parentheses. Protein purity was analyzed by SDS-PAGE. All anaerobic work was performed in MBRAUN gloveboxes ([O_2_] < 10 ppm). The synthetic cofactor [Fe_2_(μ-ADT)­(CO)_4_(CN)_2_]^2–^ or [2Fe]^ADT^ was synthesized following literature protocols with minor modifications and verified by FTIR spectroscopy.^36^ The used diquat derivatives were synthesized following literature protocols.^2,37^ The CpI encoding plasmid was kindly provided by Prof. T. Happe at the Ruhr University Bochum. Data visualization was performed using Origin 2019, R, and the tidyverse package if not stated otherwise.^38–40^

### UV–Vis Titration and Deconvolution

Titrations were conducted to measure the reduction of redox mediators and their stability over time. Spectra (230–700 nm) were recorded with 2 nm increments using a Tecan Infinite 200 Pro spectrophotometer and Magellan software in a 1 mm quartz cuvette. Titrations were performed at pH 8 with increasing terminal electron donor (Eu­(II)­EGTA or NaDT) to redox mediator ratios, with the redox mediator concentration kept constant within one measurement. Samples with varying reductant concentrations were prepared separately to minimize passive loss effects. The Eu­(II)­EGTA complex was prepared by dissolving EuCl_2_ in 0.1 M MES-HEPES pH 6 buffer (0.15 M NaCl) and EGTA in 2 M NaOH followed by mixing EuCl_2_ and EGTA (3,12-bis­(carboxymethyl)-6,9-dioxa-3,12-diazatetradecane-1,14-dioic acid) in a 1:1 ratio to a final concentration of 0.5 M.^34,35^ By using the stability constant of 9.38 for Eu­(II)­EGTA, the concentration of the formed complex was calculated as a function of pH.^41,42^ A small volume of the Eu­(II)­EGTA complex was subsequently added to samples containing the redox mediator of interest and 0.1 mM MES-HEPES buffer with the desired pH (6, 7, or 8). The pH of the sample was measured after addition to confirm that it remained at the intended value. Measurements were repeated at different redox mediator concentrations within the UV–vis absorbance window, and spectral deconvolution was performed. A sigmoidal curve was fitted to the data to quantify the fraction of the one-electron reduced redox mediator under the assay conditions employed here. See Supporting Information for more details on the deconvolution. The stability of one-electron reduced redox mediator solutions was measured for MV^•+^, DQ-H^•+^, and dmDQ-H^•+^ at pH 8, 7, and 6 with a 1:1 Eu­(II)­EGTA or 1:2 NaDT to redox mediator ratio, in triplicate over 9 min with 1.5 min intervals.

### Protein Purification and Maturation

Hydrogenase I from *C. pasteurianum* was overexpressed in *Escherichia coli* BL21­(DE3)­ΔiscR cells using a pET21b plasmid with a C-terminal StrepTag. Small cultures of 20 mL were grown overnight in LB media with ampicillin (100 μg/mL) and kanamycin (40 μg/mL) at 37 °C. Large cultures of 1 L were inoculated in LB media with 100 mM MOPS (pH 7.4), 2 mM NH_4_–Fe­(III)-citrate, 0.5% glucose, and antibiotics (ampicillin 100 μg/mL and kanamycin 40 μg/mL), grown to OD_600_ 0.4, then transferred to Schott bottles with 25 mM Na-fumarate and 1/10 dilution Antifoam C. Cultures were induced anaerobically with 5 mM l-cysteine and 0.5 mM Isopropyl β-d-1-thiogalactopyranoside (IPTG), flushed with nitrogen, and incubated at room temperature for 20 h. Cells were harvested under aerobic conditions and subsequently transferred into an anaerobic glovebox for resuspension in lysis buffer containing 100 mM Tris-HCl (pH 8.0), 150 mM NaCl, 1.2 mg/mL lysozyme, 0.06 mg/mL DNase, 0.06 mg/mL RNase, 2.4 mg/mL MgCl_2_·6H_2_O, one EDTA-free protease inhibitor tablet per 50 mL buffer, 5 mg/mL deoxycholic acid, and 5 mg/mL sucrose. After incubation and sonication, cell lysate was ultracentrifuged, filtered, and purified using a BioRad FPLC system with a StrepTrap XT column. Pure protein was flash frozen and stored at −80 °C. Protein purity was analyzed with SDS-PAGE, and concentration was determined using the Pierce BCA Protein Assay Kit. Fe content was quantified using a previously reported assay.^43^

The holo-form of CpI was obtained by incubating the apoenzyme (50 μM) with synthetic cofactor [2Fe]^ADT^ (600 μM) anaerobically at 4 °C in 100 mM NaPi buffer pH 6.8, followed by desalting with a PD-10 column using 10 mM Tris-HCl buffer pH 8.0 for elution. Holo protein was aliquoted, flash frozen, and stored at −80 °C. Concentration and iron content were measured for the purified protein (Table S1). Cofactor integration was verified using ATR-FTIR and EPR spectroscopy (see the Supporting Information, Figures S9 and S11, for more details).

### Electrolysis and Protein Film Electrochemistry

Electrolysis of dmDQ-H^2+^ to its one-electron reduced form (dmDQ-H^•+^) was performed under N_2_ in a glovebox using a three-electrode system. The system included a Ag/AgCl reference electrode (saturated KCl) and a graphite rod counter electrode, both isolated by glass frits. The working electrode was reticulated vitreous carbon foam with a thin graphite rod (2 mm diameter) inserted. The concentration of dmDQ-H^2+^ was 50 mM in 0.1 M MES-HEPES pH 8 (complemented with 0.15 M NaCl), and the total volume was 10 mL (0.5 mmol). Electrolysis continued until a total charge of −28.13 C (corresponding to the reduction of 0.292 mmol dmDQ-H^2+^ assuming 100% Faradaic efficiency) was passed since −48 C yields full conversion, and the aim was to achieve approximately 50% conversion to the one-electron reduced redox mediator (Figure S1). The end concentration was confirmed by using UV–vis spectroscopy. The solution potential of the solution post electrolysis was determined by measuring the open-circuit potential.

Protein film electrochemistry was conducted under N_2_ in a glovebox using a three-electrode system and a glass cell featuring a gas inlet for H_2_ flow control (1 atm). The system included a Ag/AgCl reference electrode (KCl saturated) and a graphite rod counter electrode, both isolated by glass frits. The working electrode was a highly oriented pyrolytic graphite rotating disk (5 mm diameter, 15 mm outer diameter PEEK shroud, PINE Research Instrumentation) rotated at 0–3000 rpm depending on the experiment. The enzyme was immobilized on the working electrode by drop casting. The buffer was 5 mM each of MES, HEPES, CHES, TAPS, and CH_3_COONa with 100 mM Na_2_SO_4_, adjusted to a desired pH by the addition of HCl or NaOH, and pH changes were achieved through buffer exchange. Currents obtained at different pH values were determined for the same protein films by comparing the average of the forward and reverse scans at a given potential.

### H_2_ Production Solution Assay

Assays were performed with 0.2 M MES-HEPES buffer (0.15 M NaCl) at pH 8, 7, and 6, and with MES-HEPES-CH_3_COONa buffer at pH 5. Fresh enzyme aliquots were used for each assay, and activity was determined through a fixed control assay (MV^2+^/Eu­(II)­EGTA at pH 8), showing a 25% variation in the activity between aliquots.

The reaction mixture was prepared in an anaerobic glovebox by diluting a 2 μL enzyme aliquot to 0.15 μM, and the enzyme solution was then further diluted to appropriate final amounts (0.2–7 pmol or 0.2–7 nM in concentration) to stay within a linear H_2_ production initial rate. Eu­(II)­EGTA was prepared fresh as a 1:1 mixture of EGTA and EuCl_2_. The formed complex concentration was calculated as a function of pH, as mentioned previously.^41,42^ A reduced redox mediator was achieved by mixing Eu­(II)­EGTA and redox mediator at a 0.9:1 ratio and NaDT at a 0.45:1 ratio. NaDT concentrations were measured at 315 nm using an extinction coefficient of 8043 M^–1^cm^–1^.^44^ Where indicated, experiments with NaDT and Na_2_SO_3_ were pH adjusted through the addition of NaOH prior to enzyme addition. In experiments with Na_2_SO_3_, Na_2_SO_3_ was added subsequent to redox mediator reduction.

Three redox mediators were used: 1,1′-dimethyl-[4,4′-bipyridine]-1,1′-diium dichloride (methyl viologen, MV^2+^), 7,8-dihydro-6H-dipyrido­[1,2-a:2′,1′-*c*]­[1,4]­diazepine-5,9-diium dibromide (diquat, DQ-H^2+^), and 3,11-dimethyl-7,8-dihydro-6H-dipyrido­[1,2-a:2′,1′-*c*]­[1,4]­diazepine-5,9-diium dibromide (dimethyl meta-substituted diquat, dmDQ-H^2+^). Reactions were initiated by adding CpI with a gastight Hamilton syringe into crimped glass vials. Initial rates were measured with increasing reduced redox mediator concentrations until saturation, except for MV^2+^ with Ti­(III)­citrate (see the Supporting Information for synthesis details), which was measured at 40 mM. Reactions were performed in 8.8 mL glass vials with a 1 mL total liquid sample volume. Initial rates were determined by measuring H_2_ formation at 90 s intervals, starting 90 s after assay initiation, using a PerkinElmer Clarus 500 gas chromatograph. Data in Figure 4 was measured using a PerkinElmer Clarus 590 gas chromatograph with a TurboMatrix 40 headspace autosampler (see the Supporting Information for instrument details). The apparent *K*_m_ and maximal turnover number of the saturation curves were determined by fitting a variant of the Hill equation to the saturation data.

$$y=\frac{k_{\mathrm{cat}}\times x^{n}}{K^{n}+x^{n}}$$

where *y* is the turnover number, *x* is the substrate concentration, *k*_cat_ is the maximum turnover rate, *K* is the apparent Michaelis constant, and *n* is the Hill coefficient.

Assays without redox mediator were performed to evaluate nonmediated electron transfer from Eu­(II)­EGTA under otherwise identical conditions to those employed with redox mediators. Initial rates were measured at three Eu­(II)­EGTA concentrations (5, 10, and 20 mM) at pH 8 with the highest used enzyme amount within the kinetic assays (7 pmol), showing a constant H_2_ formation rate of 54.7 (±12.9) s^–1^, and was deemed negligible.

## Results and Discussion

### Exploring Eu­(II)­EGTA as an Alternative Electron Donor

To identify suitable assay conditions, titration experiments were carried out with Eu­(II)­EGTA under anaerobic conditions to prevent the formation of reactive oxygen species and oxygen-induced oxidation. Here, the aim was to maximize the amount of one-electron reduced RM while minimizing the formation of the two-electron reduced form, as multiple forms of the electron donor would add complexity to the kinetic studies. Various quantities of Eu­(II)­EGTA were added to solutions with a fixed concentration of a given RM (MV, DQ-H, or dmDQ-H) in its most oxidized state (RM^2+^), and the reduction process was monitored by UV–vis spectroscopy (Figures S2–S4). Three RMs with different reduction potentials were tested: MV and the two diquats, DQ-H and dmDQ-H. Spectral deconvolution of the titration data generated for all three RMs (Figure 2A–C, top) showed that a Eu­(II)­EGTA:RM molar ratio of ≈1.1 yielded the maximal formation of the one-electron reduced species (i.e., RM^•+^), after which point the two-electron reduced redox mediator (i.e., RM ^± 0^) began to accumulate.

**Figure 2:**
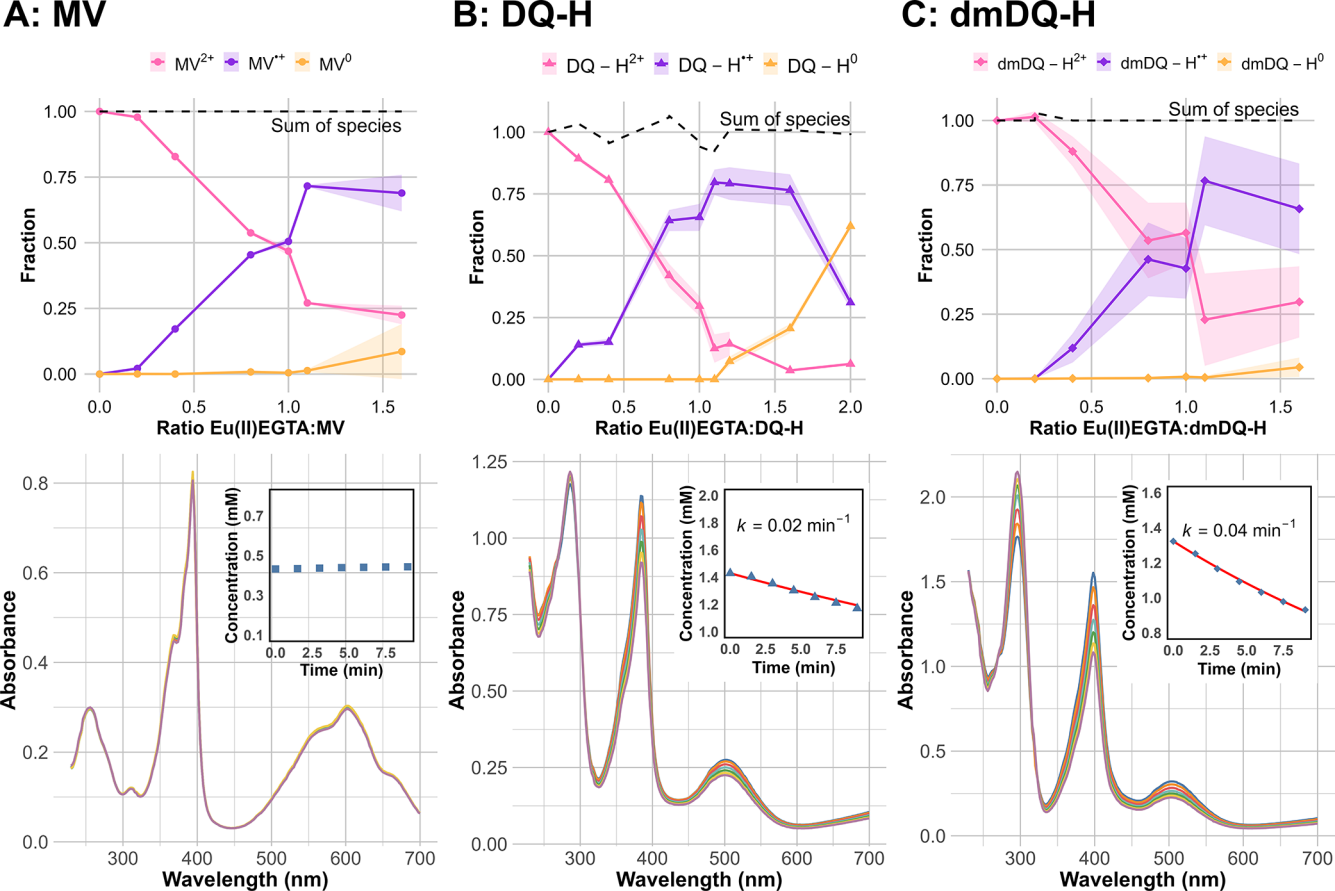
Formation (top row) and stability (bottom row) of reduced redox mediator solutions. Panels A, B, and C show data from MV^2+^, DQ-H^2+^, and dmDQ-H^2+^, respectively. (Top) Speciation data where the fractions of nonreduced RM (RM^2+^) in pink, one-electron reduced RM (RM^•+^) in violet, and two-electron reduced RM (RM^0^) in orange are plotted as a function of increasing Eu­(II)­EGTA to RM^2+^ ratio. No free Eu­(II)­EGTA absorption was observed. For details on the deconvolution of spectra, see Figures S2–S4. Each data point is plotted with a circular dot; the solid lines are plotted as a visual guide together with the standard deviation in the respective color with lower transparency. (Bottom) UV–vis spectra of pH 8 solutions containing a mixture of RM^2+/^RM^•+^, generated with a 1:1 ratio of Eu­(II)­EGTA:RM (total RM concentrations: [MV] = 0.5 mM; [DQ-H] = 2 mM; [dmDQ-H] = 2 mM). Data was collected over 9 min with 1.5 min time intervals using a 1 mm quartz cuvette. The inset shows how the concentration of the respective RM^•+^ species changes over time (monitored at 600 nm for MV^•+^ and 500 nm for DQ-H^•+^ and dmDQ-H^•+^). MV was shown to be stable, whereas DQ-H^•+^ and dmDQ-H^•+^ decrease with apparent first-order kinetics (*k*_DQ‑H_ = 0.02 and *k*_dmDQ‑H_ = 0.04 min^–1^, fits plotted as red solid line).

Additionally, the stability of the one-electron reduced RM solutions was monitored using UV–vis spectroscopy over 9 min (1.5 min intervals) at pH 6–8 with different RM concentrations. No spontaneous reoxidation of MV^•+^ was observed on the time scale of the experiment, regardless of pH (Figure 2A, bottom, only pH 8 data shown). Compared with MV, the one-electron reduced DQ-H and dmDQ-H species showed more limited stability and the reoxidation kinetics displayed a more pronounced pH dependence (Figure 2B,C, bottom). In the case of DQ-H^•+^, the reoxidation could be approximated with first-order kinetics with regard to DQ-H^•+^, and rate constants of 0.02, 0.06, and 0.13 min^–1^ at pH 8, 7, and 6, respectively, were determined (Figures S5–S7). More specifically, for a 2 mM DQ-H^2+^ solution reduced using 2 mM Eu­(II)­EGTA (≈1.4 mM DQ-H^•+^), this translates into a reoxidation rate of about 28, 84, and 182 μM DQ-H^•+^/min, corresponding to a solution potential shift of +3–28 mV in 5 min (estimated using the Nernst equation). Similarly, the spontaneous reoxidation of dmDQ-H^•+^ at pH 8 also appeared to follow first-order kinetics with regard to dmDQ-H^•+^, and a rate constant of 0.04 min^–1^ was determined (Figure S8). However, it is important to note that a rapid acceleration of the reoxidation was observed for dmDQ-H^•+^ solutions at pH 6 and 7, with a complete bleaching of the solutions observed within 10 min. The reoxidation in lower pH solutions also displayed a stronger dmDQ-H^•+^ concentration dependence as well as more complex kinetics. Thus, albeit the reoxidation was not negligible, for assays performed on a time scale of a few minutes, the combination Eu­(II)­EGTA/DQ-H can arguably be considered sufficiently stable in the complete studied pH interval. Conversely, the use of dmDQ-H^•+^ appears restricted to pH 8 under these conditions.

The Eu­(II)­EGTA:RM mixtures described above were subsequently employed for kinetic assays, using the CpI [FeFe]-hydrogenase as a model system. Based on the titration and deconvolution data, a Eu­(II)­EGTA to RM ratio of 0.9:1 was determined to be suitable for kinetic assays to ensure minimal contributions of the two-electron reduced RM to the kinetics. More specifically, addition of 0.9 mol equivalents of Eu­(II)­EGTA to the respective RM results in the formation of approximately 53% (±1), 69% (±2), and 54% (±6), respectively, of the one-electron reduced mediators, i.e., MV^•+^, DQ-H^•+^, and dmDQ-H^•+^ as calculated from a sigmoidal fit of the titration curves shown in Figure 2, top (standard deviation obtained from deconvolution). The remaining RMs reside in their respective RM^2+^ state, yielding solution potentials of ≈ −0.45, −0.57, and −0.65 V, respectively, calculated using the Nernst equation. All of the concentrations of RM^•+^ presented in the following sections have been calculated according to these expected ratios between RM^•+^ and RM^2+^.

The CpI enzyme was prepared using established protocols, with the notable omission of NaDT, and H-cluster assembly was verified through a combination of Fourier transform infrared (FTIR) and electron paramagnetic resonance (EPR) spectroscopy (Figures S9 and S11).^45^ The enzyme was isolated in a mixture of 60% H_ox_ and 40% H_ox_-CO state, estimated using EPR spectroscopy and simulations (Figure S11). As repeated thawing and freezing were found to have a very negative impact on enzyme activity, the enzyme was aliquoted before storage, and fresh aliquots were used for each assay. With every used aliquot, the activities were normalized through a fixed MV^2+^/Eu­(II)­EGTA assay performed at pH 8. The enzyme was added to preset concentrations of the one-electron reduced redox mediator under strictly anaerobic conditions, and H_2_ gas production was monitored by gas chromatography. Data was collected following a 90 s delay after assay initiation to avoid any complexity arising from mixing or enzyme activation effects. EPR spectroscopy suggests that reduction of CpI occurs on a time scale of a few seconds (Figure S12). Further, after this delay period, the influence of the original mixed H_ox_ and H_ox_-CO population on the kinetics is expected to be negligible. Once the inhibiting CO-ligand of H_ox_-CO has been released and equilibrium has been established with the headspace gas (7.8 mL), the concentration of CO in the aqueous phase (1.0 mL) is expected to be in the low pM range.^46^ The initial rate was determined within the first 5 min of the reaction, during which time the reaction rate was found to be linear (Figure S13).

The 0.9:1 molar ratio (Eu­(II)­EGTA to RM) yields a solution potential of −0.47 V for MV^2+^/MV^•+^ vs SHE, measured electrochemically with open-circuit potential measurements, in reasonable agreement with the calculated solution potential of −0.45 V. Using this fixed MV^2+^/MV^•+^ ratio, saturation curves were collected over a range of MV^•+^ concentrations for pH 6, 7, and 8 (Figure 3A).

**Figure 3:**
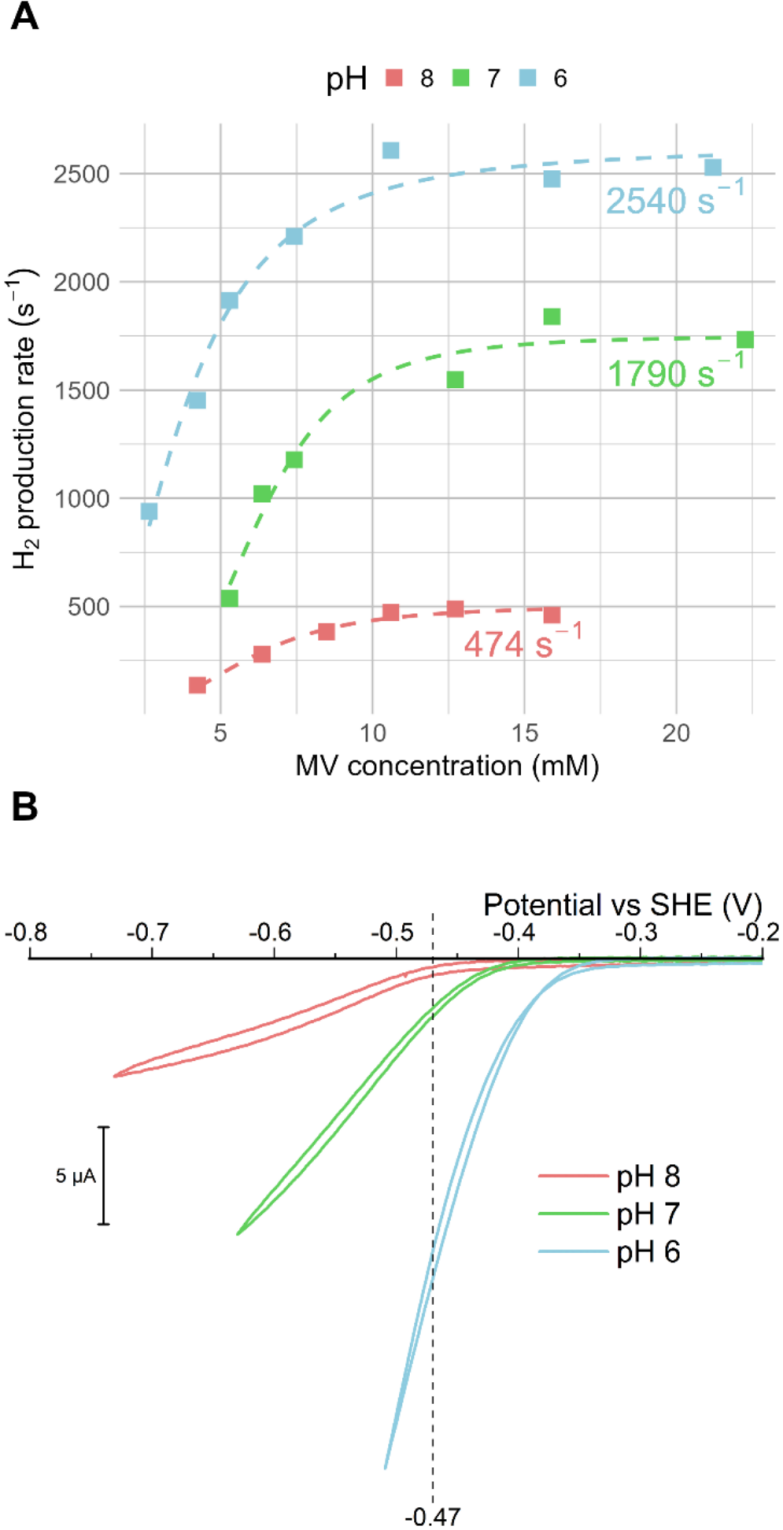
Activity data collected through solution assays (A) and protein film electrochemistry (B); color coded in light red, green, and blue for pH 8, 7, and 6, respectively. (A) Measured average rates (*n* = 3) with increasing MV^•+^ concentration at pH 8, 7, and 6. Individual data points with respective standard deviations are tabulated in Table S2. All solution assays are performed using a 200 mM MES-HEPES 150 mM NaCl buffer, and MV^•+^ was generated using Eu­(II)­EGTA. The dashed curves represent fits of the saturation equation (see Experimental Section for more details). The text annotations in the corresponding pH color indicate the obtained maximal turnover rates. (B) Cyclic voltammograms were recorded at pH 8, 7, and 6, using a rotating disc HOPG electrode with drop-casted CpI enzyme at 25 °C, 1 atm N_2_, 5 mVs^–1^ scan rate, and a rotation rate of 2500 rpm. *X*-axis shows zero current. The dashed vertical line indicates the reduction potential of the herein employed MV solution.

From this data set, the maximal obtained rate was found to increase when the pH was decreased. Compared with the maximum rate at pH 8, the rate increased 3.8-fold at pH 7 and 5.4-fold at pH 6. Qualitatively, this agrees well with what is observed with PFE (Figure 3B), as will be further discussed below. pH measurements performed after the reaction was completed showed negligible change (ΔpH < 0.1). Furthermore, to stay within the initial rate activity window, the enzyme concentration was varied between assays. Thus, the amount of formed product was consistently kept low, precluding any significant solution potential change over the course of the assay. More specifically, the largest product formation of 1160 nmol was observed with MV^•+^ at pH 7 at saturated conditions (18 mM, corresponding to a total amount of 18,000 nmol in the 1 mL assay volume, of MV^•+^), giving a solution potential change of less than 1 mV during measurements. The *K*_m_ value for MV^•+^ was determined from the saturation curves (Figure 3A) and found to be 5.9 ± 0.4 mM at pH 7 and 8, but decreased to 3.6 ± 0.2 mM at pH 6.

As the rate continuously increased down to pH 6, assays were performed also at pH 5. However, the complexation chemistry of Eu­(II) and EGTA makes it problematic to use at pH < 6.^41,42^ Thus, assays at lower pH were performed using Ti­(III)­citrate as the terminal electron donor. Control experiments showed that the rates observed at pH 6 were, within error, identical when comparing the assays with Eu­(II) or Ti­(III) as the terminal reductant (Figure 4). Similarly, no rate enhancement was observed when the pH was decreased from 6 to 5 (Figure 4). Incubating enzyme aliquots for 2–20 min at a given pH prior to initiating the assays did not result in discernible differences in final rates, which rules out pH instability as a significant factor influencing the results. In short, our results show that Eu­(II)/EGTA can be readily employed as a terminal reductant in solution assays with [FeFe]-hydrogenase and reveal that pH 5–6 is the optimal pH region for the CpI enzyme.

**Figure 4:**
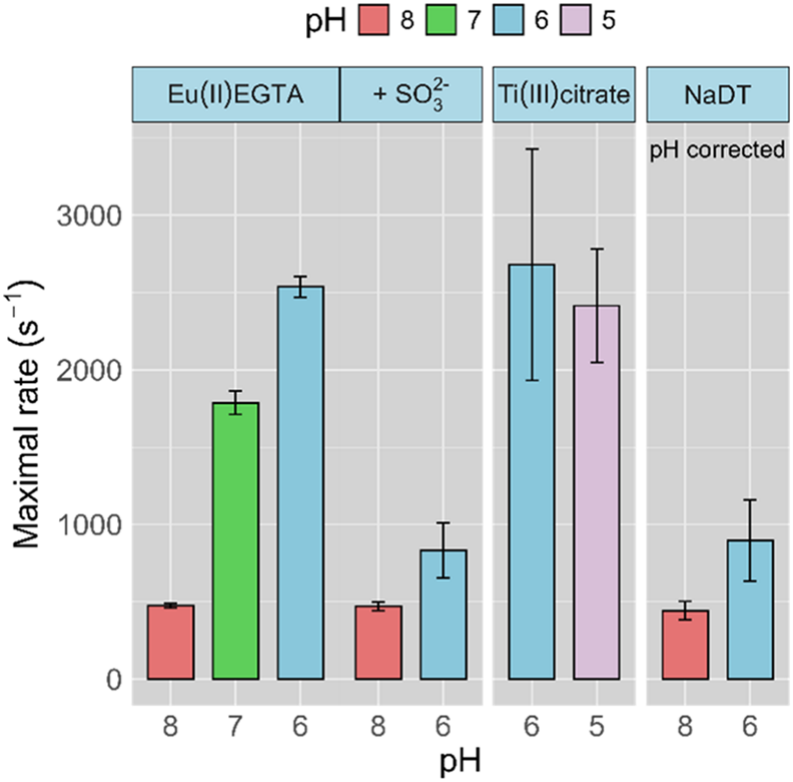
Maximum rates observed for CpI in solution assays as a function of pH and terminal reductant, using MV as RM. Activity data color coded in light red, green, blue, and light purple for pH 8, 7, 6, and 5, respectively. Average (*n* = 3) of the highest measured H_2_-evolution rate in s^–1^ of CpI using MV^•+^ at saturation (12 mM) with Eu­(II)­EGTA, Ti­(III)­citrate, or NaDT (left to right) as the terminal electron donor. Measurements with Eu­(II)­EGTA were conducted with and without the addition of 20 mM Na_2_SO_3_ at pH 8 and 6. Solution assays are performed using a 200 mM MES-HEPES 150 mM NaCl buffer; at pH 5, the buffer was complemented with acetate. The assays with Na_2_SO_3_ and NaDT were pH corrected with NaOH. Rates observed for NaDT as an electron donor without pH correction are shown in Figure S15.

For comparative purposes, the CpI enzyme was also studied using electrochemistry in the pH of range 6–8. Cyclic voltammetry traces of the CpI enzyme drop casted onto a highly oriented pyrolytic graphite (HOPG) electrode under a H_2_ atmosphere show the expected reversible catalytic behavior in good agreement with earlier reports,^33^ as reflected in clear currents attributable to H^+^ reduction and H_2_ oxidation evident already with minor deviation from the thermodynamic potential (Figure S14). To facilitate the comparison between the solution assay and electrochemical measurements, cyclic voltammetry traces were recorded under a N_2_ atmosphere (Figure 3B). Although care should be taken when interpreting absolute currents in PFE, the effect of pH can be estimated by comparing the currents obtained for the same film as the pH of the buffer is varied. Comparing currents observed at −470 mV (vs SHE), an approximate 4- and 25-fold increase was observed for pH 7 and 6, respectively, relative to the currents observed at pH 8. A direct comparison between PFE and solution experiments is challenging due to enzyme heterogeneity arising from surface immobilization, but the pH trend aligns with both previously published data for CpI (see, e.g., ref 36) as well as the results of the solution assays described above.

### Effect of NaDT and SO_3_^2–^ on Solution Assays

To compare and contrast Eu­(II)­EGTA with NaDT, assays with NaDT were performed analogously to those using Eu­(II) or Ti­(III), although the stoichiometry of terminal electron donor to redox mediator was calculated taking into consideration that NaDT is a two-electron donor. Titration experiments were conducted in a similar manner as with Eu­(II)­EGTA, and all presented MV^•+^ concentrations are reported based on the observation that our specific assay conditions (a ratio of 0.45:1 of NaDT to MV^2+^) yield approximately 63% (±2) one-electron reduced MV (MV^•+^) (Figure S17). This gives a calculated solution potential of −0.46 V, that is, 10 mV more positive compared with the potential measured for Eu­(II)­EGTA reduced MV, which may contribute to minor variations in activity and should be considered when interpreting the effects of NaDT. As with MV and Eu­(II)­EGTA, no reoxidation of MV^•+^ was observed within a 9 min time frame (Figure S18).

Two issues became evident with the use of NaDT from these studies: pH drift and apparent enzyme inhibition. While pH remained stable in the case of Eu­(II) and Ti­(III), a significant acidification was observed with NaDT upon reduction of MV^2+^. This observation is readily explained by the fact that oxidation of dithionite results in the release of two molar equivalents of protons, effectively decreasing the pH of the solution as the reduction of MV^2+^ and/or CpI progresses.^23,25^ Within the saturation curve collected in a pH 8.0 buffer (Figure S15), with the identical buffer composition as used for Eu­(II), the pH decreased to 7.6 when 10 mM MV^2+^ was reduced with NaDT. An even larger decrease, to pH 7.3, was observed when 30 mM MV^2+^ was reduced. Therefore, the data points with NaDT and MV^•+^ depicted in Figure S15 do not reflect a true representation of the saturation curve as the pH changes with MV^•+^, and, by extension, NaDT concentration. Still, a *K*_m_ value of 4.3 ± 0.4 mM can be approximated. At lower pH, no saturation curve was observed; this could be a result of the pH shift induced by increasing NaDT concentration, an inhibitory effect of NaDT, or a combination of both. In short, in our assays, NaDT generates multifaceted data, making a single unequivocal interpretation difficult. For a more direct comparison to Eu­(II)­EGTA data, measurements were also performed with pH-adjusted solutions, generated through the addition of NaOH subsequent to NaDT-induced reduction of MV^2+^. Such measurements were performed at saturated conditions (12 mM MV^•+^), and data at pH 8 were shown to be in agreement with the rates measured with Eu­(II)­EGTA as the terminal electron donor. However, the rate enhancement observed at pH 6 was significantly smaller with NaDT than what was obtained for Eu­(II)­EGTA (Figure 4).

The differences in rate at lower pH between the two terminal reductants are striking. As the MV^•+^ and MV^2+^ concentrations and ratio are highly similar between the two assays it confirms that NaDT, or its oxidation products, indeed has an inhibitory effect on [FeFe]-hydrogenase, as previously proposed by Martini et al.^24^ As noted above, the oxidation product sulfite is in equilibrium with the bisulfite ion (HSO_3_^–^, p*K*_a_ = 7.19), which in turn participates in an equilibrium with sulfur dioxide (SO_2_ (*aq*) + H_2_O (*l*) ⇌ H^+^ (*aq*) + HSO_3_^–^ (*aq*) p*K*_a_ = 1.86).^47^ Inhibition by SO_2_ could thus rationalize the observed pH dependent divergence between the two terminal reductants. Although we note that this would require a very high affinity for the inhibitor, the expected SO_2_ concentration is in the nM range even at pH 6. Our results show that, at pH 6, the rate obtained with NaDT as the terminal electron donor is 3-fold lower than the rate obtained with Eu­(II)­EGTA at the same pH value. At lower pH, the formation of SO_2_ in the assay increases, and thereby the inhibition. To further solidify this hypothesis, sodium sulfite (Na_2_SO_3_) was added to kinetic assays, employing Eu­(II)­EGTA as the terminal electron donor. The obtained rates at pH 8 and 6, with 12 mM MV^•+^ (MV^•+^ saturated conditions), show rates that are within error of those obtained with NaDT as the terminal electron donor when 20 mM Na_2_SO_3_ is added to the reaction (Figure 4). Thus, the inhibitory effect of NaDT oxidation products on [FeFe]-hydrogenase, possibly in combination with pH drift, is highly likely to explain the discrepancies in previously published H_2_ evolution kinetics reported for CpI. For example, a study by Erbes et al. showed a continuous increase of the maximum rate down to pH 7.0 (the lowest measured pH value in the latter study).^22^ Conversely, Duan et al. have reported a pH optimum of 8.0, whereas Adams et al. have reported a pH optimum of 6.3 with a significant decrease of activity at pH 5.9 and lower.^1,48^ All three studies use MV^2+^ as redox mediator in combination with NaDT and none share an agreement with published PFE characterization^33^ or the data obtained in our Eu­(II)­EGTA/Ti­(III)­citrate assays.

SO_2_-inhibition has been proposed to give rise to an H-cluster species denoted as H_ox_H (also referred to in some literature as H_ox_-DT_i_), with a clearly distinguishable FTIR spectroscopic signature.^24,49^ Our spectra obtained during the reduction of NaDT free CpI with Eu­(II)­EGTA were highly similar to those previously reported for HoxH in CpI, although a shift of the bridging CO band by about 13 cm^–1^ was observed relative to the H_ox_H signature observed with NaDT and Na_2_SO_3_ (Figure S10). The origin of this latter shift is currently unknown, but this H_ox_H-like spectroscopic signature has been observed also when producing the H_ox_H state in *Chlamydomonas reinhardtii* HydA1 using DTT as reductant.^50^ Thus, although kinetic differences are evident between assays using Eu­(II) or NaDT as terminal reductants, FTIR spectroscopy suggests that highly similar H-cluster states are formed.

### H_2_ Evolution Rates of CpI at Different Solution Potentials

As seen in Figure 3B, the currents recorded during cyclic voltammetry do not reach a steady-state value as the potential is swept in the negative direction but rather display a continuous increase in reductive current as the driving force increases. The same trend has been observed also in earlier PFE studies of CpI and other [FeFe]-hydrogenases.^8,33,51–55^ This effect is commonly attributed to variations in interfacial electron transfer distances due to a disordered orientation of the adsorbed protein molecules onto the electrode, resulting in a dispersion of interfacial electron transfer rates.^56,57^

By using other redox mediators, DQ-H and dmDQ-H (Figure 1), the catalytic activity of the enzyme spanning a larger potential window could now be studied also in solution.^2,3^ The obtained solution potentials, determined with open-circuit potential measurements, using the aforementioned Eu­(II)­EGTA:RM molar ratio of 0.9:1, were −0.56 V for DQ-H^2+^/DQ-H^•+^ and −0.65 V for dmDQ-H^2+^/dmDQ-H^•+^ vs SHE, reflecting an increase of 90 and 180 mV, respectively, relative to the MV assays (−0.47 V for MV^2+^/MV^•+^). Critically, the use of DQ derivatives enabled these studies while remaining under saturating conditions with regard to the RM concentrations.

Saturation curves were determined with DQ-H^•+^ in the pH range of 6–8 (Figure 5A), revealing a *K*_m_ of 8.9 ± 0.7 mM at pH 7 and 8. As for MV^•+^, a slight decrease of the *K*_m_ was observed at pH 6, but here the difference was smaller and within the error margins of the measurements at higher pH (*K*_m_ at pH 6 = 8.3 ± 0.3 mM). The data also show that the trend of increasing activity with decreasing pH remains with DQ-H^•+^ as the redox mediator (Figure 5B), albeit less pronounced compared with MV^•+^. With DQ-H^•+^, rates increase by a factor of 1.2 for pH 7 and 1.4 for pH 6, relative to pH 8. The kinetic assays with dmDQ-H^•+^ were restricted to pH 8, due to the stability issues noted above, and a *K*_m_ of 11.7 ± 1.2 mM was determined.

**Figure 5:**
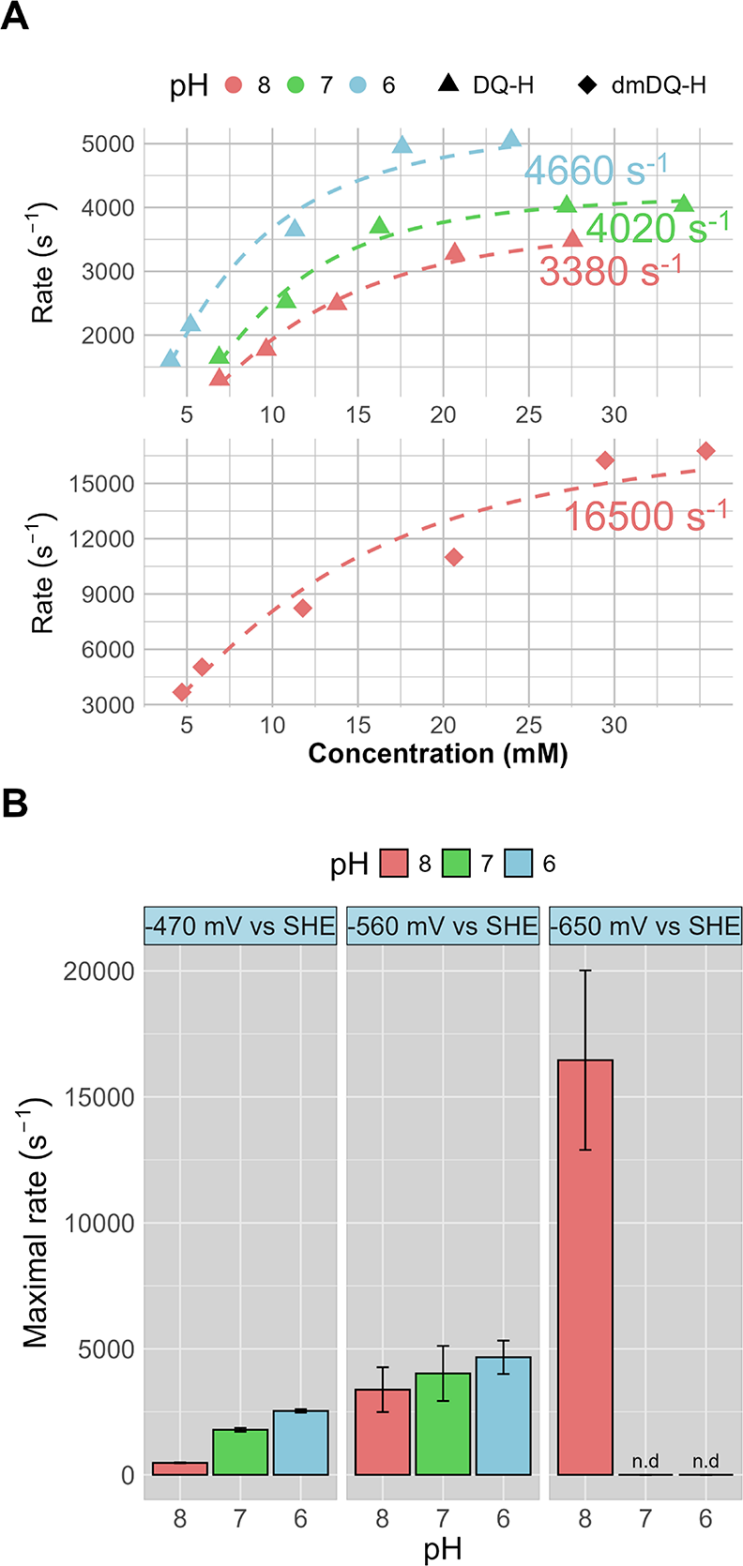
Saturation curves (A) and maximum rates (B) observed for CpI as a function of pH and the solution potential. Activity data color coded in red, green, and blue for pH 8, 7, and 6, respectively. (A) Measured average rates (*n* = 3) with increasing RM^•+^ concentrations for DQ-H (triangle shape) and dmDQ-H (diamond shape). The dashed curves represent fits of the saturation equation (see Experimental Section for more details). The text annotations in the corresponding pH color indicate the obtained maximal turnover rates. (B) Bar chart of measured maximal rate in s^–1^ at three different solution potentials (−470, –560, and –650 mV vs SHE) set by using three different redox mediators (MV, DQ-H, and dmDQ-H). Rates with dmDQ-H at pH 7 and 6 were not determined (see the main text) and are labeled “n.d.” Error bars represent the standard deviation. All solution assays are performed using 200 mM MES-HEPES and 150 mM NaCl buffer with Eu­(II)­EGTA as the terminal electron donor.

In addition to the clear effect of pH, a strong correlation between the rates and solution potential was observed when MV^2+^ was replaced by DQ-H^2+^ and dmDQ-H^2+^ (Figure 5B). Comparing the maximum H_2_ production rates, relative to MV^2+^/MV^•+^, the increase of solution potential to −0.56 V (DQ-H^2+^/DQ-H^•+^) resulted in a 7-fold increase in the rate at pH 8 (Figure 5B). The highest maximal rate was observed with dmDQ-H^•+^ as the redox mediator, where a solution potential of −0.65 V yielded a TOF of 16500 ± 3600 s^–1^ (Figure 5B), corresponding to a 35-fold increase relative to assays employing MV^•+^ at the same pH. The higher maximal rates likely contribute to the somewhat higher *K*_m_ values observed with the diquats compared with MV. In contrast, the decrease in *K*_m_ values at lower pH may reflect changes in binding affinity. In order to confirm that the rate enhancement was not connected to the presence of Eu­(II), analogous assays were performed using the previously defined dmDQ-H^2+^/dmDQ-H^•+^ ratio generated through bulk electrolysis. No significant difference in rate was observed compared with that in assays employing Eu­(II)­EGTA (Figure S19). The solution potential dependence observed here thus differs from the earlier report by van Dijk et al. for the [FeFe]-hydrogenase from *Megasphaera elsdenii*,^20^ in that it is evident across a much larger potential window. If this difference reflects intrinsic differences between the two enzymes remain to be verified, as it could potentially arise from variations in the assay methodology (e.g., single-concentration point analysis and using NaDT as terminal reductant, vs full saturation curves with Eu­(II)­EGTA as terminal reductant).

The rate enhancements observed in solution assays, such as the 35-fold increase from MV to dmDQ-H at pH 8, significantly exceed those predicted from the Marcus theory. This assumes similar reorganization energies and electronic coupling for all three redox mediators with the only major difference being the change in driving force. Under these assumptions, the measured increase in rate with increasing driving force implies an average reorganization energy, calculated from the ratios of rate constants, of 0.5 eV for MV to dmDQ-H at pH 8, and between 0.16 and 0.24 eV for MV to DQ-H across pH 6–7. These values are significantly lower than the expected total system reorganization energy of ≥2.24 eV, estimated as the sum of reported values for Fe–S and H-clusters of hydrogenases (≥1.7 eV)^58^ and for the mediators themselves (0.54–0.68 eV).^59^ Even using the lower combined reorganization energy value of 2.3 eV, Marcus theory would predict a rate increase only up to 7-fold when proceeding from MV to dmDQ-H, rather than the observed 35-fold. An extended discussion on the Marcus theory analysis is provided in the Supporting Information (see Supporting Note 1). Considering the comparable *K*_m_ values for MV^•+^ and the diquats, a similar affinity and binding for the redox mediators is likely, but the complexity of the *K*_m_ parameter prevents a definitive statement. However, comparing rates under saturated conditions does allow for the investigation of solution potential dependence without interference from redox mediator binding contributing to the overall kinetics. We tentatively attribute the strong solution potential dependence to a complex interplay between the H-cluster and the electron relay F-clusters although other factors may certainly be involved, such as structural changes close to the H-cluster.^13,60,61^ Further, the apparent decrease in pH dependence observed in solution assays as the solution potential becomes more negative suggests a switch in mechanism under more reducing conditions. We speculate that sufficiently reducing potentials shifts the mechanism away from a more energy-efficient but relatively slow process involving concerted proton–electron transfer. Instead, electron transfer may be accelerated to the point where it starts to become disconnected from proton transfer steps; follow-up protonation of the prereduced H-cluster is then also expected to occur at high rates. With this being said, additional data are clearly needed to substantiate this claim. Here, it should also be noted that the CV traces retain a stronger pH dependence also at more negative potentials (Figure 3), evident from comparing both absolute currents and the difference in slope of the current relative to applied potential at different pH. The latter observations imply that proton transfer kinetics has a higher influence across a wider potential range under PFE conditions. This difference between solution assays and PFE will require further studies to clarify but could reflect differences in electron injection routes and/or differences in pH dependence in binding affinities to the electron donors (MV, DQ, electrode surface).

## Conclusions

In summary, this study outlines a new solution assay for probing the enzymatic activity under reducing conditions. It has been demonstrated that Eu­(II)­EGTA is a robust reductant in the pH range of 6–8. Moreover, at least in the case of [FeFe]-hydrogenase, the presence of Eu­(II)­EGTA did not appear to alter the kinetics of the enzyme, as exemplified by two different redox mediators. The same rates as for Eu­(II)­EGTA were observed with dmDQ-H^•+^ produced electrochemically and MV^•+^ produced using Ti­(III)­citrate. We also note that the observed rates using dmDQ-H are, within the margin of error, the same as the rate previously reported for the [FeFe]-hydrogenase from *Ca*HydA (21000 ± 12000 s^–1^), determined through extrapolation using single-molecule imaging.^30^ However, we stress that the TOF of ≈16500 ± 3500 s^–1^ observed here still remains a lower limit of the actual TOF, as further rate enhancement is likely achievable with optimization of, for example, buffer composition and additives. In terms of mechanistic information, the new solution data on kinetics reveals that CpI does in fact display a distinct solution potential dependence. This represents a key insight for future kinetic modeling of data acquired from protein film electrochemistry and other techniques. Furthermore, the assays employed herein reveal a lower pH optimum for CpI than generally observed in NaDT-dependent solution assays. Critically, using Eu­(II)­EGTA in place of NaDT as the terminal reductant now results in a clearer correlation between PFE and solution assay data with regard to pH dependence. Here, we have limited the assay to the well-studied model enzyme CpI, but we predict that similar trends will be observed also for many other [FeFe]-hydrogenases, while stressing that variations are likely considering the diverse nature of this enzyme family. We also note that kinetic information derived from solution assays under saturated conditions is not convoluted by the effects arising from the binding of redox mediators, enabling more valid comparisons between experimental conditions. The discrepancy between NaDT and other terminal electron donors studied here (Eu­(II)­EGTA, Ti­(III)­citrate, and the electrode surface) is attributable to a range of factors arising from the complex pH dependent speciation of NaDT and its oxidation products. Specifically for [FeFe]-hydrogenase, a key issue is the inhibitory effect observed at low pH, evident from comparing assay data generated using NaDT and Eu­(II)­EGTA. The observation that the inhibition appears at low pH and can be replicated through the addition of SO_3_^2–^ to the Eu­(II)­EGTA-based solution assay strongly supports the hypothesis of SO_2_-induced enzyme inhibition proposed by Martini et al. Conversely, our FTIR data do not fully support the notion that SO_2_ inhibition is reflected in the state referred to as H_ox_H as an H_ox_H-like H-cluster state was observed also with Eu­(II)­EGTA.^24^ More broadly, the assay methodology reported herein is expected to be applicable for the study of other related (gas-processing) redox enzymes. It overcomes several of the issues associated with NaDT and provides a convenient entry point for studies of driving force dependence when combined with suitable redox mediators.

## Supplementary Material



## Notes

Additional experimental methods for UV–vis spectral deconvolution, ATR-FTIR spectroscopy, EPR spectroscopy, H_2_ calibration curve generation, and Ti­(III) citrate synthesis. It also includes supplementary results and figures from protein purification, bulk electrolysis of dmDQ-H, Eu­(II)­EGTA titrations, rate constant determinations, and cofactor integration evaluations using ATR-FTIR and EPR spectroscopy. Additionally, it covers the linearity of initial rate measurements, NaDT reduced MV saturation curves, titrations and deconvolution with NaDT, stability measurements, control experiments, initial rates, extinction coefficients and spectra from deconvolution for MV, DQ-H and dmDQ-H species, H_2_ calibration curves, and the UV–vis spectrum of synthesized Ti­(III) citrate. An extended discussion on Marcus theory analysis is also included (PDF)

## ⊥

Fakultät für Chemie and Biochemie, Anorganische Chemie I, Ruhr-Universität Bochum, Universitätsstraße 150, 44801 Bochum, Germany

## Abbreviations

ADT: azadithiolate
CaHydA: [FeFe]-hydrogenase from *Clostridium acetobutylicum*
CpI: [FeFe]-hydrogenase I from *Clostridium pasteurianum*
dmDQ-H: dimeta-methyl-substituted diquat
DQ-H: unsubstituted diquat
EPR: electron paramagnetic resonance
FTIR: Fourier transform infrared
HOPG: highly oriented pyrolytic graphite
*K*_m_: Michaelis constant
MV: methyl viologen
NaDT: sodium dithionite (Na_2_S_2_O_4_)
PFE: protein film electrochemistry
RM: redox mediator
SHE: standard hydrogen electrode
TOF: turnover frequency
