# Supplementary material for: Reconsidering the Enzyme Kinetics of [FeFe]-Hydrogenases: Improved Turnover Rates and New Insights into pH and Potential Dependence with Eu(II)-Based Solution Assays
Source: Anal Chem. 2025 Nov 25;97(48):26393–403. doi: 10.1021/acs.analchem.5c02898 (PMC12874204; doi:10.1021/acs.analchem.5c02898)
Supplement: Supplementary file 1 [file ac5c02898_si_001.pdf]

Supplementary information

Reconsidering the Enzyme Kinetics of [FeFe]-Hydrogenases: Improved Turnover Rates and New Insights into pH and Potential Dependence with Eu(II)-Based Solution Assays

Eda Sönmez[a], Nikolaos Kostopoulos[a], Mira Gamache[a,†], Mun Hon Cheah[a], Ping Huang[a], Andrew J. Bagnall[a], Dawit T. Filmon[b], Ivan Voloshyn[d], Thomas Happe[c], Moritz Senger[d], Nicolas Plumeré[b], Alina Sekretareva[a], Gustav Berggren[a, *]

Institutions

[a]Department of Chemistry - Ångström, Molecular Biomimetics, Uppsala University, Box 523, 75120 Uppsala, Sweden; E-mail: Gustav.berggren@kemi.uu.se

[b]TUM Campus Straubing for Biotechnology and Sustainability - Technical University of Munich, Uferstrasse 53, 94315 Straubing, Germany

[c]Department of Plant Biochemistry, Faculty of Biology and Biotechnology, Photobiotechnology, Ruhr University Bochum, Universitätsstrasse 150, 44801, Bochum, Germany

[d]Department of Chemistry - BMC, Biochemistry, Uppsala University, Box 523, 75120 Uppsala, Sweden

[†] Current address: Fakultät für Chemie und Biochemie, Anorganische Chemie I, Ruhr-Universität Bochum, Universitätsstraße 150, 44801 Bochum, Germany

Contents

Additional Experimental Details S3
UV-Vis spectra deconvolution S3
ATR-FTIR spectroscopy S3
Transmission FTIR spectroscopy S3
EPR spectroscopy S4
H_2 calibration curve S4
Ti(III) citrate synthesis S5
Supplementary figures and results S5
Protein stock concentration and Fe-content quantification S5
Bulk Electrolysis of dmDQ-H^{2+} S6
Titration with Eu(II)EGTA S7
Rate constant determination of spontaneous re-oxidation of $RM^{\bullet+}$ S9
FTIR spectroscopy S11
EPR spectroscopy S13
Initial rates of solution assays S15
PFE under H_2 atmosphere S16
Saturation curve of NaDT reduced methyl viologen S17
Methyl viologen with NaDT titration and deconvolution S18
NaDT reduced methyl viologen stability measurements S19
Control experiments to determine the effects of addition of acetate or Ti(III)citrate S20
Tabulated initial rates S21
Obtained spectra from deconvolution S22
H_2 calibration curves S23
Spectrum of Ti(III)citrate S24
Supplementary notes S24
Supplementary note 1. Extended discussion on Marcus theory analysis S24
References S26

Additional Experimental Details

UV-Vis spectra deconvolution

Deconvolution was done on spectra obtained with increasing Eu(II)EGTA to redox mediator ratios. Reference spectra of each unreduced redox mediator, annotated RM^{2+} (MV^{2+}, DQ-H^{2+}, dmDQ-H^{2+}), Eu(II) and Eu(III) were recorded individually. The spectrum of each one-electron reduced redox mediator ($RM^{•+}$) was obtained by subtracting the RM^{2+}, Eu(II) and Eu(III) spectra from spectra where Eu(II)EGTA and respective redox mediator were present until the bands attributed to RM^{2+}, Eu(II) and Eu(III) were as close to zero as possible. This was done for the two lowest Eu(II)EGTA to redox mediator ratios (0.2:1 and 0.4:1 Eu(II)EGTA to redox mediator) to avoid the presence of two-electron reduced redox mediator. This deconvolution was repeated twice with $RM^{•+}$ reference spectra: first for the 0.2:1 ratio, and then for the 0.4:1 ratio, in order to determine the standard deviation associated with the method. Spectra for each two-electron reduced redox mediator were obtained in the same way as for each one-electron reduced redox mediator, with the additional step of subtracting the $RM^{•+}$spectrum, extracted from the ratio measurements with the highest Eu(II)EGTA concentration (1.6:1 for MV^{2+} and dmDQ-H^{2+} and 2:1 for DQ-H^{2+} Eu(II)EGTA to redox mediator). The obtained spectra of the different species and their calculated extinction coefficients are shown in Figure S20.

Deconvolution of each spectrum was performed in Microsoft Excel, where the spectrum from each Eu(II)EGTA:RM^{2+} ratio is modelled as a linear combination of the spectra of RM^{2+}, $RM^{•+}$, $RM^{••}$, Eu(II)EGTA and Eu(III)EGTA.

$$\begin{aligned} Spectrum\ of\ mixture \\ &= n_1(ref.\ spectrum\ of\ RM^{2+}) + n_2(ref.spectrum\ of\ RM^{•+}) \\ &+ n_3(ref.spectrum\ of\ RM^{••}) + n_4(ref.spectrum\ of\ Eu^{II}) \\ &+ n_5(ref.spectrum\ of\ Eu^{III}) \end{aligned}$$

The values of n (representing the relative population of each species) were determined by the least squares fitting method, using the solver function in Microsoft Excel.

ATR-FTIR spectroscopy

Concentrated CpI protein of 1 µL (50 mg/mL) in 10 mM Tris-HCl buffer pH 8 was pipetted on the ATR crystal under anaerobic atmosphere. The ATR unit (BioRadII, Harrick) was sealed with a PEEK cell (custom build)[1] and mounted within a FTIR spectrometer (Vertex V70v, Bruker). The sample was dried using 100% N_2-gas, as described before.[1] The spectra were recorded with a scanner velocity of 80 Hz and 2 cm^{-1}. All measurements were performed at room temperature and atmospheric pressure.

Transmission FTIR spectroscopy

A solution of 4 µL of concentrated CpI protein (73 mg/mL) in 10 mM Tris-HCl buffer pH 8 was mixed with 0.6 µL 250 mM Eu(II)EGTA in 200 mM MES-HEPES (150 mM NaCl) pH 6 buffer and deposited on the CaF_2 window in the anaerobic Ar atmosphere of a Vigor glove box. The sample was sealed with a second CaF_2 window and mounted on a custom build transmission cell which allowed for gas exchange and illumination. The cell was installed in a FTIR spectrometer (Vertex ISF66v, Bruker). The sample was dried under 100% N_2-gas and

rehydrated with a humidified aerosol (100 mM Bis-Tris, pH 6) similar to the method previously reported for our ATR-FTIR spectroscopy setup.[1] Spectra were recorded with 1 cm^{-1} resolution, a scanner velocity of 80 Hz and averaged of varying number of scans (typically 100 scans). All measurements were performed at ambient conditions (room temperature and pressure). Gas (N_2) was applied at a flow rate of 1.5 L/min with varying humidity following our established protocol for $H_{ox}H$ population.[2]

EPR spectroscopy

EPR spectra on the "as isolated" and reduced CpI samples (Figure S11 and S12) were collected on a Bruker EMX-micro spectrometer equipped with an EMX-Premium bridge generating X-band microwave. An ER4119HS resonator was connected with an Oxford Instruments continuous flow cryostat for low temperature operating. Measuring temperatures were achieved using liquid helium flow through an ITC 503 temperature controller (Oxford Instruments).

EPR samples with reduced CpI were prepared by adding a small volume (5.5 µL) of enzyme to EPR tubes and thereafter adding a larger volume (74.5 µL) containing buffer with or without reduced redox mediator to the small volume to mix before flash freezing the samples after approximately 5 s. This was done to ensure that the reaction had not proceeded too far before freezing the samples. Six samples were prepared with 50 µM enzymes in each and with two different redox mediators (10 mM) at pH 7. One sample was prepared with only 50 µM enzyme in buffer pH 8 and a blank EPR sample for background noise control was prepared containing buffer (200 mM MES-HEPES 150 mM NaCl pH 8).

H_2 calibration curve

Calibration curves were generated on two different gas chromatograph instruments: PerkinElmer Clarus® 500 and PerkinElmer Clarus®590. The Clarus® 500 contained a thermal conductivity detector and a molecular sieve packed (60/80) column (stainless steel). The temperature settings were 100°C, 80°C and 100 °C for injection port, the oven and the detector, respectively. Argon was used as carrier gas with a flow rate of 35 mL min^{-1}. The Clarus® 590, combined with a TurboMatrix 40 headspace sampler, contained a thermal conductivity detector and the gas separation was done on a hayesep N 60/30 (NR021501) and a molecular sieve 13x (NR022501). The autosampler provides programmed pneumatic control with high pressure sampling. The temperature setting was 110°C, 60°C and 200°C for injection port, the oven and the detector, respectively. Argon was used as carrier gas with a flow rate of 30 mL min^{-1}. To convert the observed H_2-integrals into amounts of H_2 a calibration curve was determined (Figure S21-S22). Glass crimp vials (8.8 mL) were filled with 1 mL MES-HEPES buffer and sealed within a glovebox. Five standards were prepared with H_2 amount ranging from 0.01 atm to 1 atm. From these standards 10 samples were prepared (~100-26000 ppm) by replacing different volumes of gas in the glass crimp vials from different standards using a gastight Hamilton syringe. The samples were placed on a shaker for 10 min before sampling 100 µL of the headspace, using a gastight Hamilton syringe, via gas chromatography. Every concentration was prepared and measured twice. The expected amount in each sample was calculated using the Ideal Gas Law (Eq. 1):

$$n(H_2) = \frac{p}{RT} \times \frac{V_{ppm}}{V_{headspace}} \qquad \text{Eq. 1}$$

Where n is the amount of H_2 in mol, p is the pressure (101325 Pa), R is the ideal gas constant (8314.463 $L{\cdot}Pa{\cdot}K^{-1}{\cdot}mol^{-1}$), T is the temperature (293.15 K), V_{ppm} is the added amount of H_2 to the sample and $V_{headspace}$ is the volume of the headspace when the vials contain 1 mL solution (7.8 mL).

The obtained integrals were thereafter plotted vs the calculated amounts of H_2 in the samples and a linear regression was performed with the y-intercept fixed to 0. The obtained linear fit was thereafter used to convert the H_2 peak integrals to amounts by following Eq. 2:

$$n_{total}(H_2) = A_{obs} \times slope \qquad \text{Eq. 2}$$

Ti(III) citrate synthesis

Due to the limited volume of the gas chromatography vials, it was necessary to prepare concentrated solutions of the reducing agents, which could effectively reduce the entire sample even with small injection volumes. Therefore, previous literature protocols[3-5] were adapted to produce a solution of approximately 0.27 M titanium(III) citrate, using commercial 10–15% titanium(III) chloride in 12% hydrochloric acid (Sigma-Aldrich) as the titanium source to mitigate the risk of solid Ti(III) chloride reacting violently with moisture/air. To our knowledge, this is the highest concentration at which aqueous titanium(III) citrate has been reported for use as a reducing agent.

Under nitrogen, 2.0 mL of the purple commercial titanium(III) chloride solution (~ 0.3 g, 2 mmol, 1 M) was injected dropwise into a pre-purged 2.0 mL aqueous solution of trisodium citrate (1.03 g, 4.00 mmol, 2.0 M, 2 eq.). Some fuming was observed, followed by a change in color to very dark violet. Following this, a pre-purged solution of sodium carbonate (1.27 g, 12.0 mmol, 3.0 M, total volume 4.0 mL) was gradually injected into the reaction mixture until bubbling ceased, indicating neutralization of the acid, with no further change in appearance. This occurred after injection of roughly 3.3 mL (~5 eq.) carbonate solution. The concentrated titanium(III) citrate solution was aliquoted into oxygen-free sealed crimp vials and stored in the glovebox for 14h. The concentration of titanium(III) citrate was determined using the previously reported extinction coefficient at 340 nm of 0.73 $mM^{-1}cm^{-1}$ (Figure S23).[4]

Supplementary figures and results

Protein stock concentration and Fe-content quantification

Table S1. Measured protein concentration and iron content of used holo-form stock solution

Protein stock concentration	Fe/protein
47 ± 1.6 mg/mL	18.5 ± 0.2

Bulk Electrolysis of dmDQ-H^{2+}

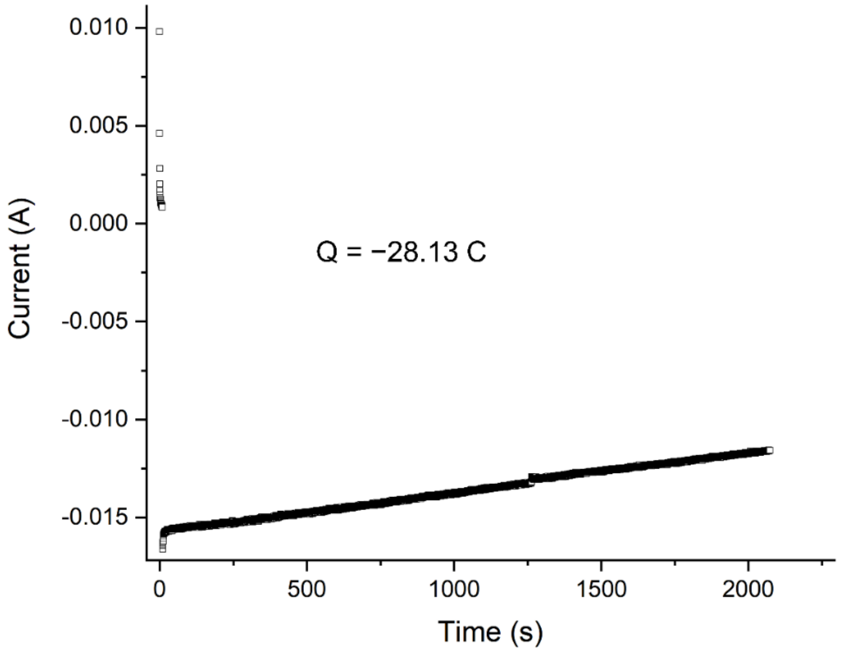

Figure S1. Bulk electrolysis of dmDQ-H^{2+}. A potential of −0.910 mV vs SHE was applied on a homemade electrolytic cell, the working electrode being a carbon foam. Concentration of the mediator was 50 mM in 200 mM MES-HEPES 150 mM NaCl pH 8 buffer, and the total volume was 10 mL. A stirring bar was used to provide continuous mixing. The electrolysis was carried out for 40 minutes and the total charge passed was −28.13 C while a full conversion of the redox mediator to its reduced form would require 48 C assuming 100% Faradaic efficiency. The open circuit potential (OCP) was measured after electrolysis and was determined to be −641 mV vs SHE (in agreement with dmDQ-H^{2+}/ dmDQ-$H^{\bullet+}$ ratio measured with UV-Vis spectroscopy). The solution potential thus displayed a ~9 mV difference with Eu(II)EGTA reduction assay.

Titration with Eu(II)EGTA

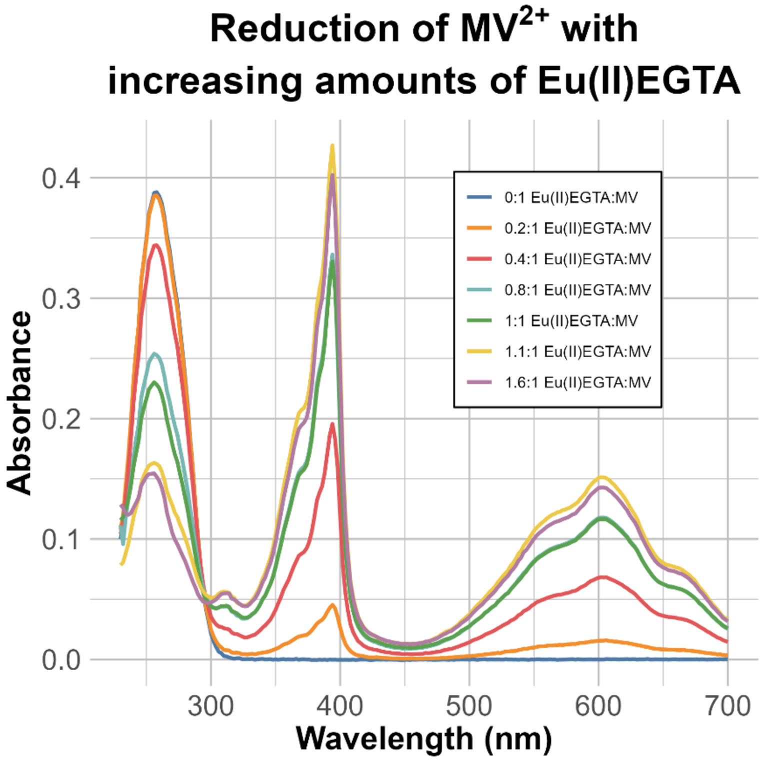

Figure S2. Titration/reduction of MV^{2+} with Eu(II)EGTA followed by UV/Vis spectroscopy. Example spectra of increasing Eu(II)EGTA to MV^{2+} ratio using a 0.1 cm quartz cuvette. Measured with 0.25 mM MV^{2+} and varying concentration of Eu(II)EGTA (0-0.40 mM). Similar results were obtained with different MV^{2+} concentrations between 0.25-0.5 mM range (studied range). The highest $MV^{•+}$ peaks were observed when the Eu(II)EGTA to redox mediator ratio was 1.1:1. The obtained spectra were used for deconvolution (Figure 2).

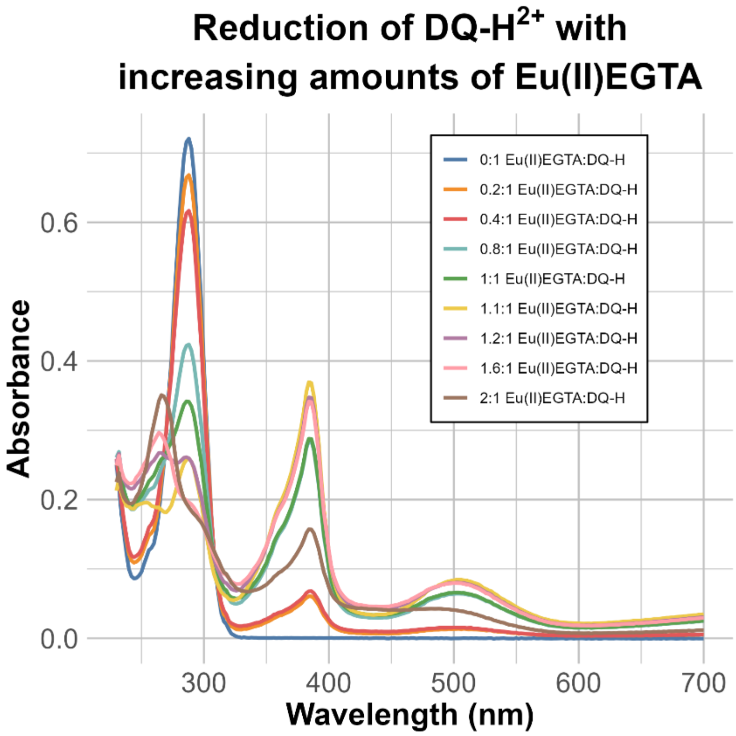

Figure S3. Titration/reduction of DQ-H^{2+} with Eu(II)EGTA followed by UV/Vis spectroscopy. Example spectra of increasing Eu(II)EGTA to DQ-H^{2+} ratio using a 0.1 cm quartz cuvette. Measured with 0.50 mM DQ-H^{2+} and varying concentration of Eu(II)EGTA (0-1.00 mM). Similar results were obtained with different DQ-H^{2+} concentrations between 0.25-5 mM range (studied range). The highest DQ-H$^{\bullet+}$ peaks were observed when the Eu(II)EGTA to redox mediator ratio was 1.1:1. The obtained spectra were used for deconvolution (Figure 2).

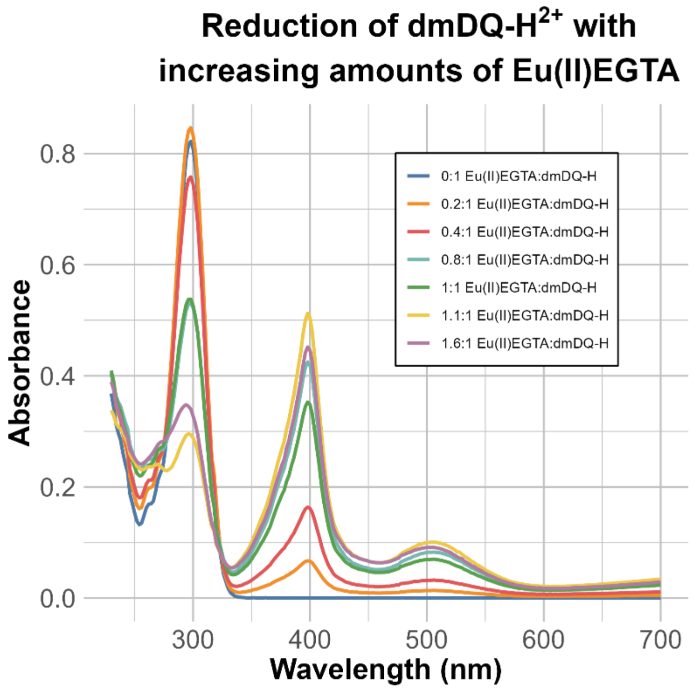

Figure S4. Titration/reduction of dmDQ-H^{2+} with Eu(II)EGTA followed by UV/Vis spectroscopy. Example spectra of increasing Eu(II)EGTA to dmDQ-H^{2+} ratio using a 0.1 cm quartz cuvette. Measured with 0.50 mM dmDQ-H^{2+} and varying concentration of Eu(II)EGTA (0-0.80 mM). Similar results were obtained with different dmDQ-H^{2+} concentrations between 0.25-5 mM range (studied range). The highest dmDQ-H$^{\bullet+}$ peaks were observed when the Eu(II)EGTA to redox mediator ratio was 1.1:1. The obtained spectra were used for deconvolution (Figure 2).

Rate constant determination of spontaneous re-oxidation of $RM^{•+}$

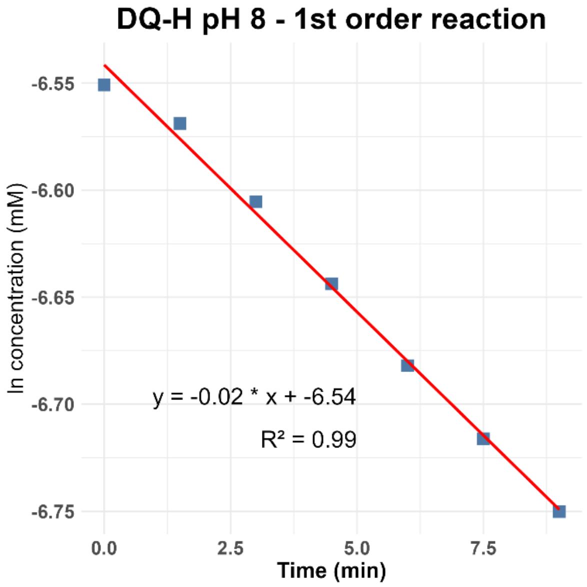

Figure S5. Plot of ln [DQ-H$^{•+}$] vs time at pH 8. Natural logarithm of the concentration of DQ-H$^{•+}$ (2 mM) decaying as a function of time shown in blue squares. Linear fit (red) is performed to determine the rate constant (k) of the 1st order reaction. A rate constant of 0.02 min^{-1} was determined with an R^2-value of 0.99.

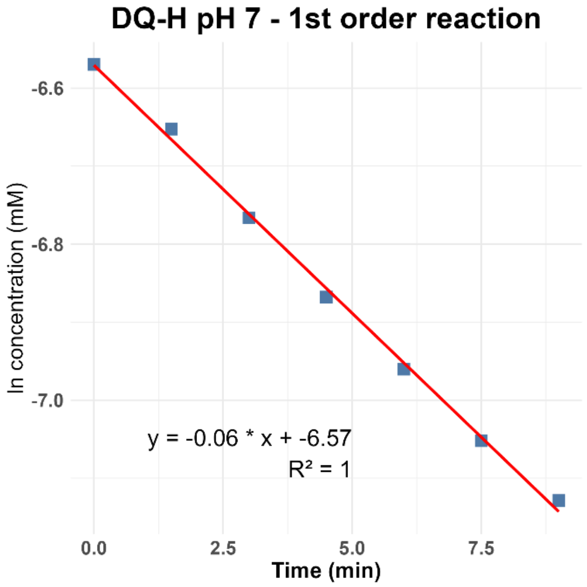

Figure S6. Plot of ln [DQ-H$^{•+}$] vs time at pH 7. Natural logarithm of the concentration of DQ-H$^{•+}$ (2 mM) decaying as a function of time shown in blue squares. Linear fit (red) is performed to determine the rate constant (k) of the 1st order reaction. A rate constant of 0.06 min^{-1} was determined with an R^2-value of 1.00.

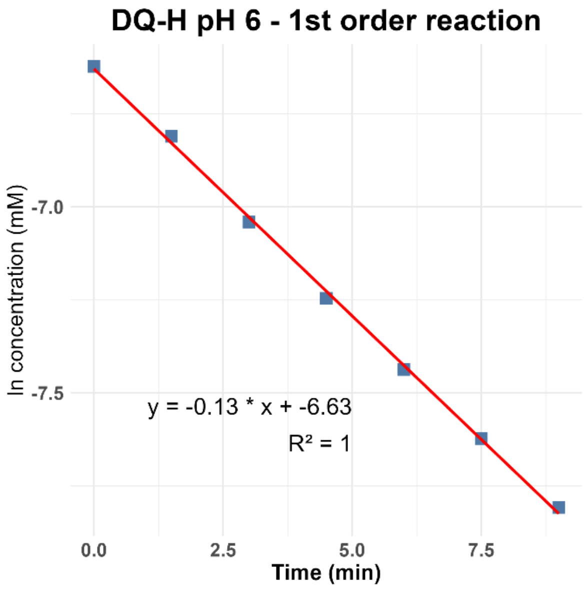

Figure S7. Plot of ln [DQ-H$^{•+}$] vs time at pH 6. Natural logarithm of the concentration of DQ-H$^{•+}$ (2 mM) decaying as a function of time shown in blue squares. Linear fit (red) is performed to determine the rate constant (*k*) of the 1st order reaction. A rate constant of 0.13 min^{-1} was determined with an R^2-value of 1.00.

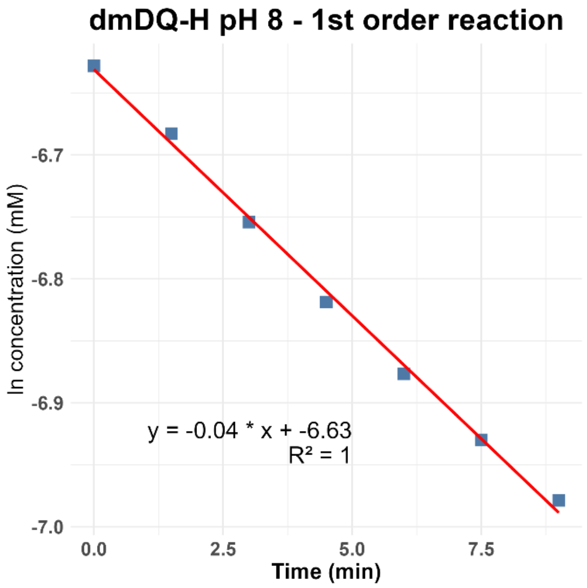

Figure S8. Plot of ln [dmDQ-H$^{•+}$] vs time at pH 8. Natural logarithm of the concentration of dmDQ-H$^{•+}$ (2 mM) decaying as a function of time (9 min, 1.5 min intervals) shown in blue squares. Linear fit (red) is performed to determine the rate constant (*k*) of the 1st order reaction. A rate constant of 0.04 min^{-1} was determined with an R^2-value of 1.00.

FTIR spectroscopy

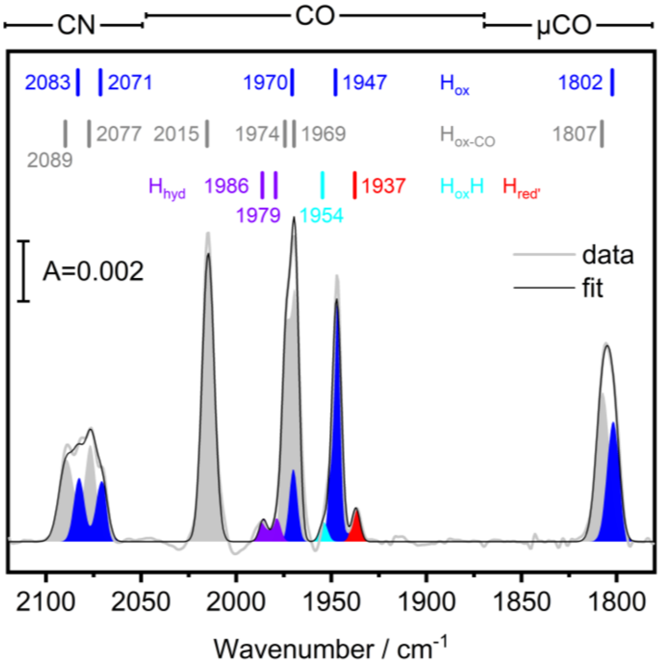

Figure S9. ATR-FTIR spectra of holo-CpI to confirm cofactor integration. ATR-FTIR spectrum of CpI [FeFe]-hydrogenase as isolated. The redox state populations are mainly the oxidized state (H_{ox} in blue) and the CO inhibited state (H_{ox}-CO in grey) with traces of the [4Fe4S] cluster reduced state (H_{red}' in red), the potentially protonated oxidized state (H_{ox}H in cyan) and the terminal hydride state (H_{hyd} in purple).

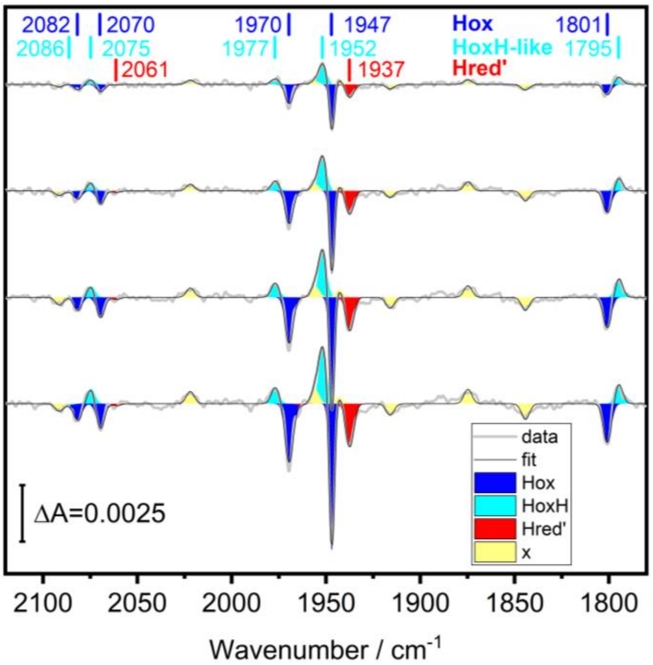

Figure S10. Transmission FTIR difference spectra of holo-CpI in the presence of Eu(II)EGTA. Transmission-FTIR spectra of NaDT-free holo-CpI in the presence of 32.6 mM Eu(II)EGTA. The differences shown report on different hydration levels of the sample. As previously shown, lowering the water content in the protein film (equivalent to an increase in concentration of reductant) facilitates the population of $H_{ox}H$.[2] The CpI film was partially dehydrated (from top to bottom) and differences were computed versus a more hydrated state of the protein film. Cyan bands indicative of a $H_{ox}H$-like state rise, while bands indicative of H_{ox} (blue) and H_{red}’ (red) decrease. Unassigned peaks are marked in yellow. Note that band position of the bridging CO ligand is downshifted by 13 cm^{-1} while all other bands are shifted by a maximum of 2 cm^{-1} compared to $H_{ox}H$.[6] Control experiments showed that FTIR spectra of our CpI preparations collected at pH 6 in the presence of either Na_2SO_3 or NaDT resulted in formation of a species with a spectroscopic signature in good agreement to that reported for $H_{ox}H$ in CpI treated with NaDT, i.e. with bridging CO ligand signal appearing at 1808 cm^{-1} (data not shown).[6, 7]

EPR spectroscopy

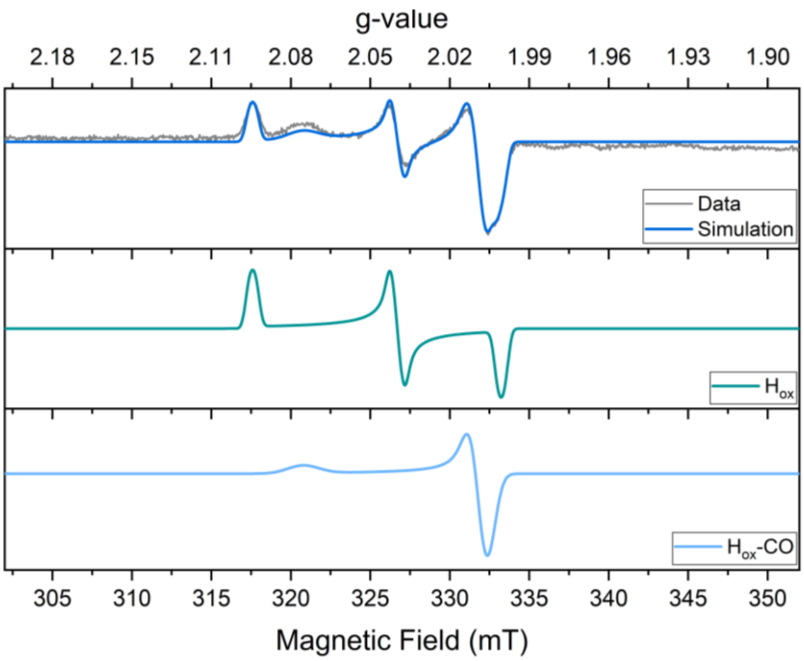

Figure S11. EPR-spectrum of as isolated CpI enzyme. EPR-spectrum of 50 µM CpI enzyme as isolated (data in grey and simulation in dark blue) in 200 mM MES-HEPES 150 mM NaCl pH 8 buffer to evaluate cofactor integration. T: 18 K; microwave frequency 9.31 GHz, microwave power 16 µW, modulation frequency 100 MHz, modulation amplitude 10 G. Two distinct states were observed H_{ox} and H_{ox}-CO. The simulations show that approximately 60% reside in the H_{ox} state (cyan spectrum, g-values 2.10, 2.04, 2.00) and 40% in the H_{ox}-CO state (light blue spectrum, g-values 2.08, 2.01, 2.01), the g-values obtained from the simulations are in agreement with published g-values.[8]

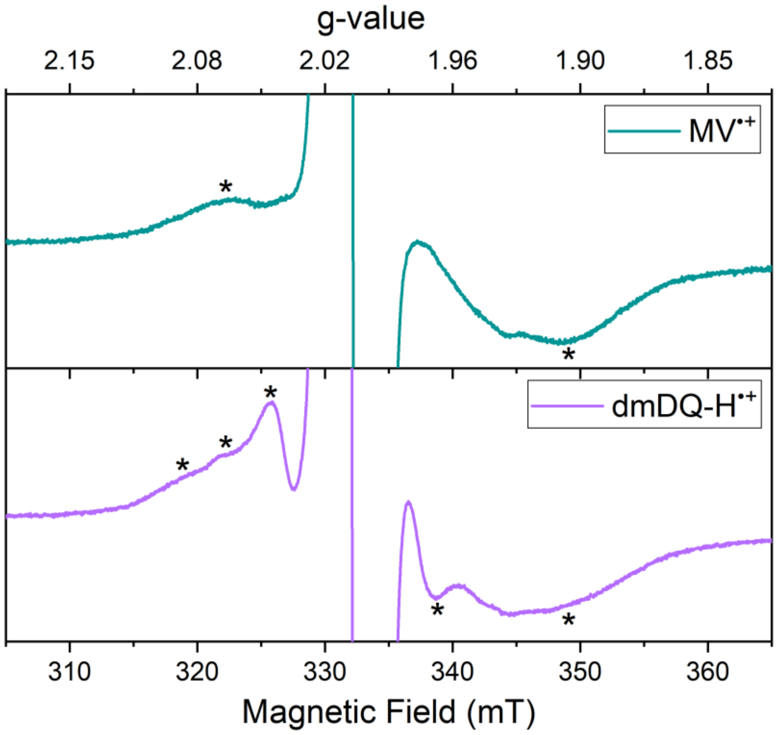

Figure S12. EPR-spectrum of CpI enzyme under turnover condition. EPR spectra collected of 50 µM enzyme reduced with 10 mM $MV^{•+}$ (**top**, dark cyan spectrum) or 10 mM dmDQ-$H^{•+}$ (**bottom**, purple spectrum) in 200 mM MES-HEPES 150 mM NaCl pH 7 buffer. Samples were flash frozen in liquid nitrogen approximately 5 seconds after enzyme and reductant mixing. Spectra recorded at 15 K; microwave frequency 9.37 GHz, microwave power 400 µW, modulation frequency 100 MHz, modulation amplitude 10 G. Interpretation of the spectra is complicated by the presence of a strong radical signal attributed to the presence of $RM^{•+}$ (truncated signal at g ≈ 2.0). Given the complexity of the system, considering the presence of radical species at high concentrations and the possible interference between all of the magnetic species residing in such a non-steady-state system, a fitting was not attempted. Still, the loss of H_{ox} on short seconds time-scale is evident from the absence of a signal component at g=2.10. Further, the appearance of reduced F-clusters is clearly indicated by the emergence of new features in the spectrum located in the wings of the radical signal. Tentative peak and trough positions are marked with asterisks as a visual guide.

Initial rates of solution assays

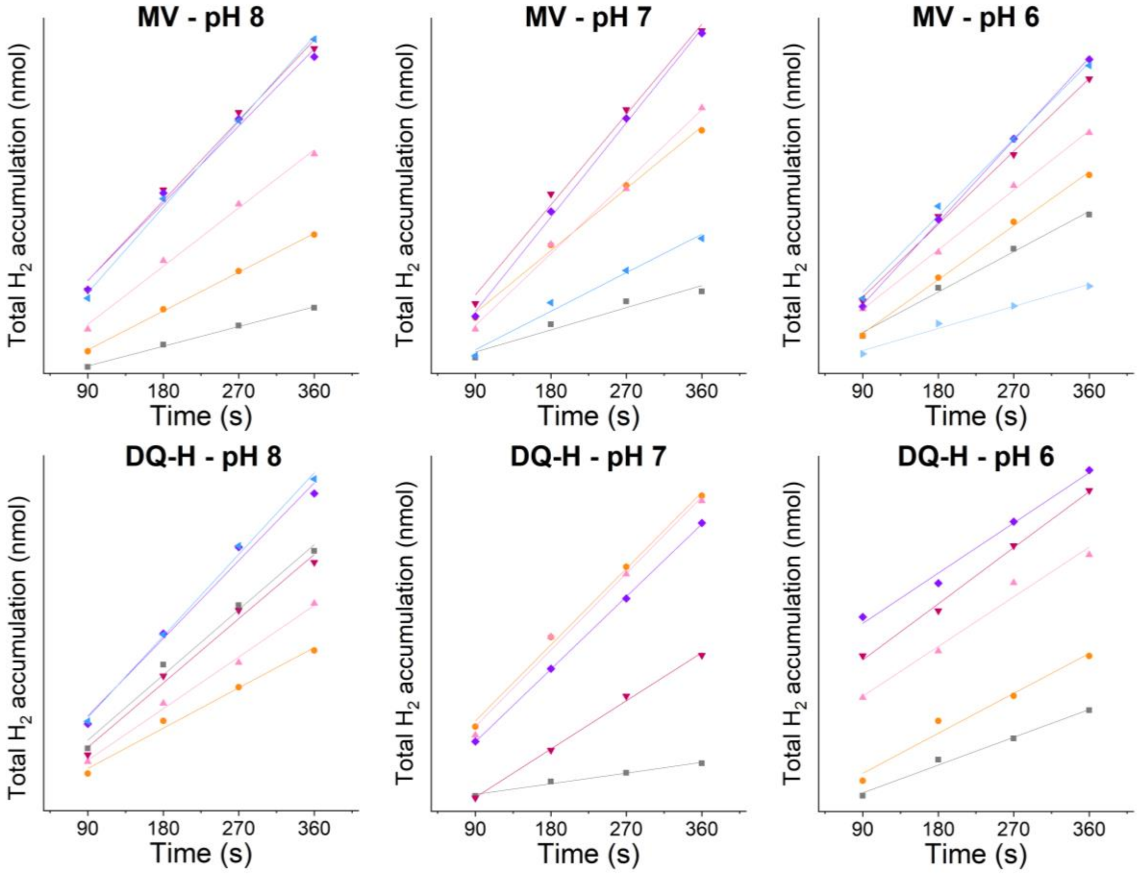

Figure S13. Measured initial rates. Shows linearity of initial rates (slope) for $MV^{\bullet+}$ and DQ-$H^{\bullet+}$ (Eu(II)EGTA reduced) over eight minutes. The colors grey, orange, light pink, dark pink, purple, blue and black are shown for increasing concentrations of each one-electron reduced redox mediator ($RM^{\bullet+}$). In each case, only one out of three replicates are shown for easier visualization.

PFE under H_2 atmosphere

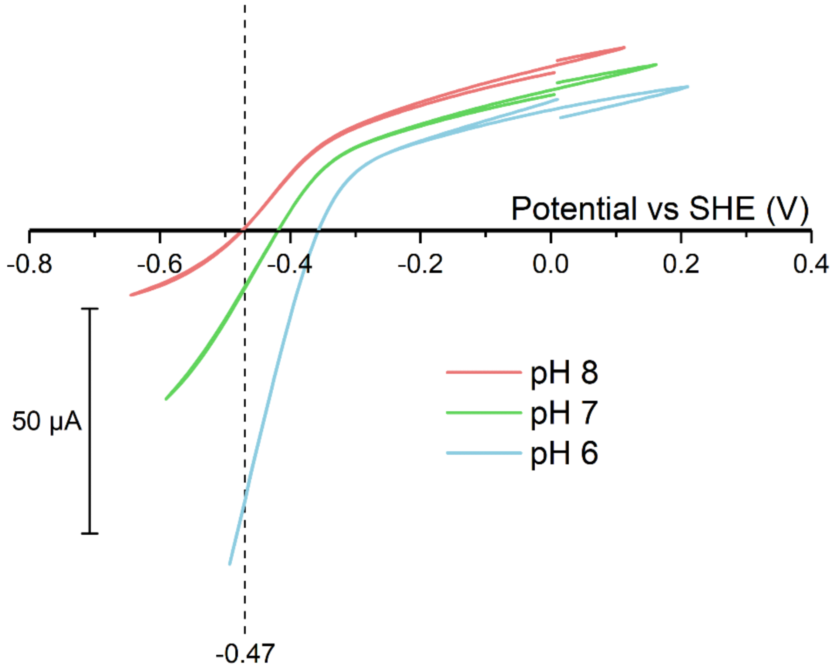

Figure S14. Protein film electrochemistry under H2 atmosphere. Cyclic voltammograms recorded at pH 8 (red), pH 7 (green), and pH 6 (blue), using a rotating disc (HOPG) electrode under 1 atm H_2. Measurements were conducted at a scan rate of 5 mV s^{-1} and a rotation speed of 2500 rpm. The CpI enzyme was drop-cast onto the electrode surface, and all experiments were performed at 25°C. The x-axis is centered at zero current, and the dashed vertical line denotes the reduction potential of the methyl viologen (MV) solution used in this study.

Saturation curve of NaDT reduced methyl viologen

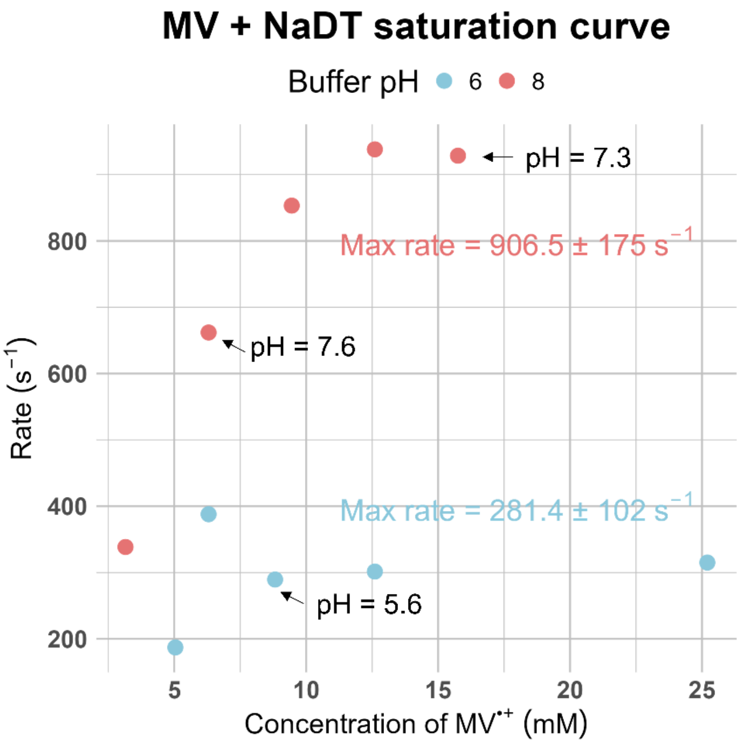

Figure S15. Saturation curve of NaDT reduced MV. Measured rates with increasing one-electron reduced MV ($MV^{\bullet+}$) concentration with NaDT as the terminal electron donor. Assays were performed in 200 mM MES-HEPES, 150 mM NaCl pH 8 (red) and pH 6 (blue) buffer. Color coding reflects starting buffer pH-values, not the actual pH during measurements. pH-values indicated in the plot reflect actual pH-values determined at a given $MV^{\bullet+}$ and NaDT concentration. Increasing $MV^{\bullet+}$, and by extension NaDT, concentration led to a gradual pH decrease: at the start of the pH 8 saturation curve (~6 mM) a pH of 7.6 is measured whereas at saturation (~16 mM) the pH was 7.3. Similar effects were observed with NaDT at lower pH where the pH was measured to be 5.6 at ~9 mM $MV^{\bullet+}$. No clear saturation curve was observed at pH 6. The same buffer system was used in the assays with NaDT and Eu(II)EGTA).

Methyl viologen with NaDT titration and deconvolution

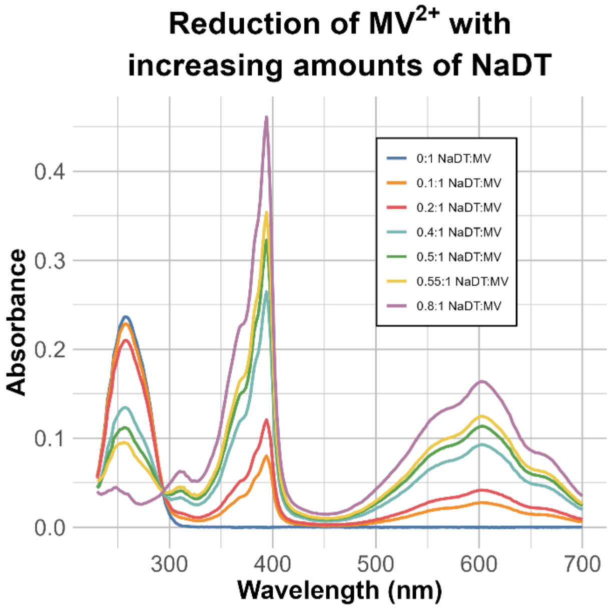

Figure S16. Titration/reduction of MV^{2+} with NaDT followed by UV/Vis spectroscopy. Example spectra of increasing NaDT to MV^{2+} ratio mixed with increasing concentrations of NaDT using a 0.1 cm quartz cuvette. Measured with 0.25 mM MV^{2+} and varying concentration of NaDT (0-0.20 mM). Similar results were obtained with different MV^{2+} concentrations between 0.25-0.5 mM range (studied range). Obtained spectra were used for deconvolution (Figure 2).

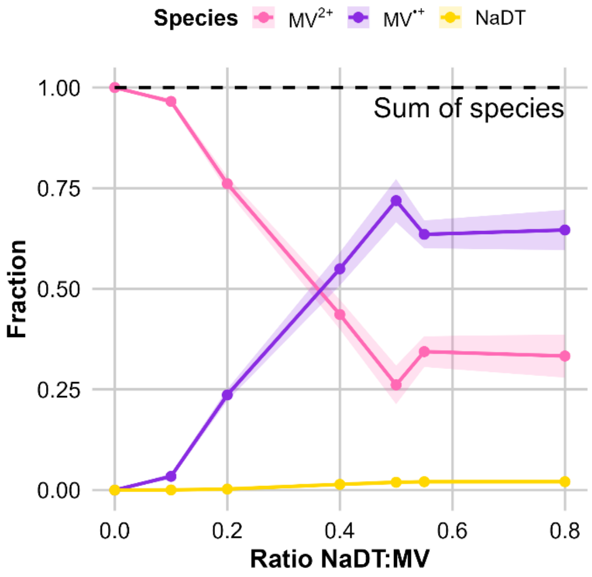

Figure S17. Deconvolution of MV reduced with NaDT. Deconvolution data shows the fractions of non-reduced MV (MV^{2+}) in pink, one-electron reduced MV (MV^{+}) in violet and NaDT in yellow. In contrast to what was observed with Eu(II)EGTA no significant amount of two-electron reduced species (MV^{0}) was observed at high NaDT to MV ratios. The average of the deconvolution is shown in straight line (as visual guide) with circular symbols for each data point of the measured ratios together with the standard deviation in respective color with lower transparency.

NaDT reduced methyl viologen stability measurements

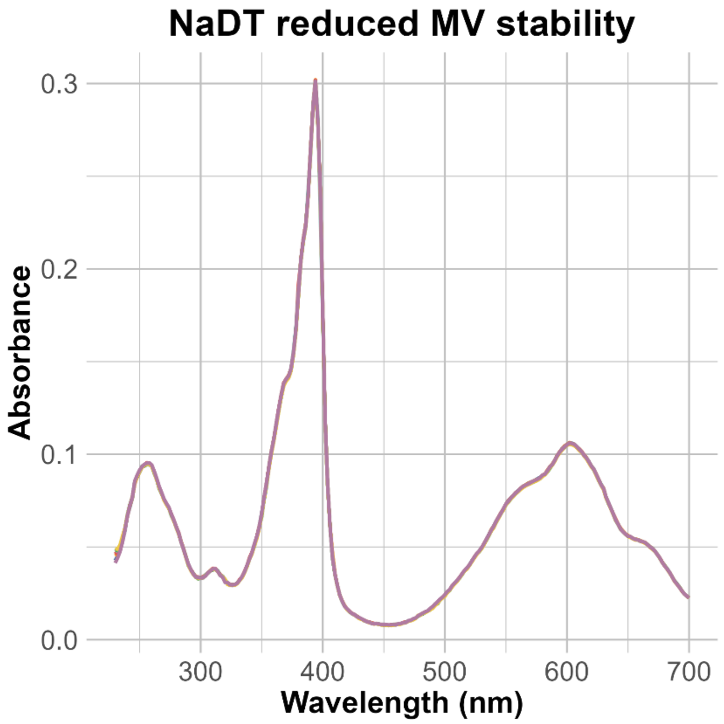

Figure S18. Stability of NaDT reduced MV. UV-Vis spectra of 0.25 mM MV^{2+} reduced with 0.13 mM NaDT (i.e. yielding 0.18 mM $MV^{\bullet+}$) collected over nine minutes at pH 8 with 1.5 min time intervals using a 0.1 cm quartz cuvette. No variation in concentration of MV species was observed within the monitored time period.

Control experiments to determine the effects of addition of acetate or Ti(III)citrate

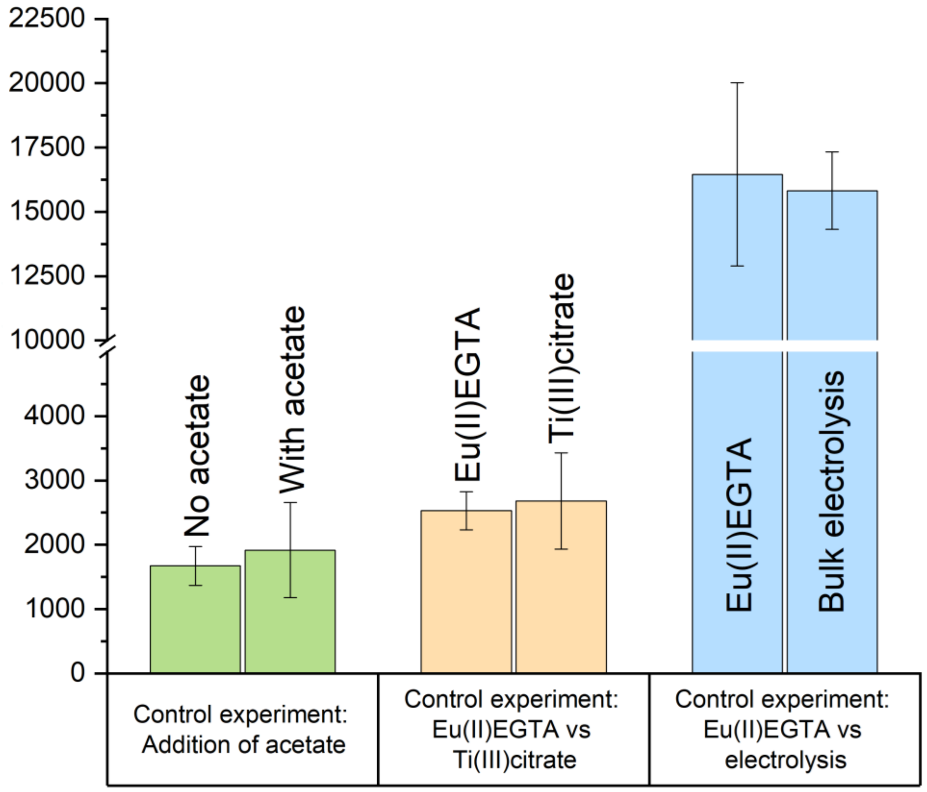

Figure S19. Grouped bar chart of measured H_2-evolution rates in control experiments. Green bars show measured H_2-evolution rates with 10 mM $MV^{\bullet+}$ (with Eu(II)EGTA) at pH 6 with 200 mM MES-HEPES 150 mM NaCl or 200 mM MES-HEPES-Acetate 150 mM NaCl buffer to determine whether buffer composition has an effect on the activity. Orange bars show measured H_2-evolution rates with 40 mM Eu(II)EGTA or Ti(III)citrate reduced MV^{2+} at pH 6 to determine whether there is a significant change in rate between the two different reducing agents. Measurements at pH 5 required acetate to be present in the buffer for buffering capacity and Ti(III)citrate to be used as an alternative reducing agent to circumvent the poor Eu(II)EGTA complex formation at lower pH values. Blue bars show measured H_2-evolution rates at saturated conditions with Eu(II)EGTA or electrochemically one-electron reduced dmDQ-H^{2+}. Errors bars show the standard deviation (n = 3). No significant difference was observed for any of the control experiments. Note the break in the Y-axis.

Tabulated initial rates

Table S2. Tabulated initial rates. Measured initial rates of H_2 evolution (divided by used enzyme concentration) in s^{-1} with increasing one-electron reduced (Eu(II)EGTA as terminal electron donor) redox mediator concentration in mM.

Redox mediator ($RM^{•+}$)	pH	Concentration of $RM^{•+}$ in mM	Rate divided by [E] in s^{-1}	Standard deviation
**Methyl viologen ($MV^{•+}$)**	**8**	4.2	136	71
		6.4	280	52
		8.5	383	156
		10.6	473	64
		12.7	489	77
		15.9	460	110
	**7**	5.3	537	91
		6.4	1020	99
		7.4	1178	26
		12.7	1547	274
		15.9	1840	13
		22.3	1733	90
	**6**	2.7	936	275
		4.2	1452	262
		5.3	1914	272
		7.4	2210	329
		10.6	2607	268
		15.9	2474	90
		21.2	2529	23
**Diquat (DQ-$H^{•+}$)**	**8**	6.9	1307	279
		9.6	1775	228
		13.8	2489	202
		20.7	3276	978
		27.6	3481	823
	**7**	6.9	1648	484
		10.8	2520	766
		16.3	3690	646
		27.2	4016	543
		34.1	4031	70
	**6**	4.0	1606	126
		5.2	2157	597
		11.3	3643	1014
		16.7	4949	748
		24.0	5052	672
**Dimethyl meta-substituted diquat (dmDQ-$H^{•+}$)**	**8**	4.7	3670	115
		5.9	5034	1243
		11.8	8232	1938
		20.6	10996	2194
		29.5	16253	4667
		35.4	16763	2631

Obtained spectra from deconvolution

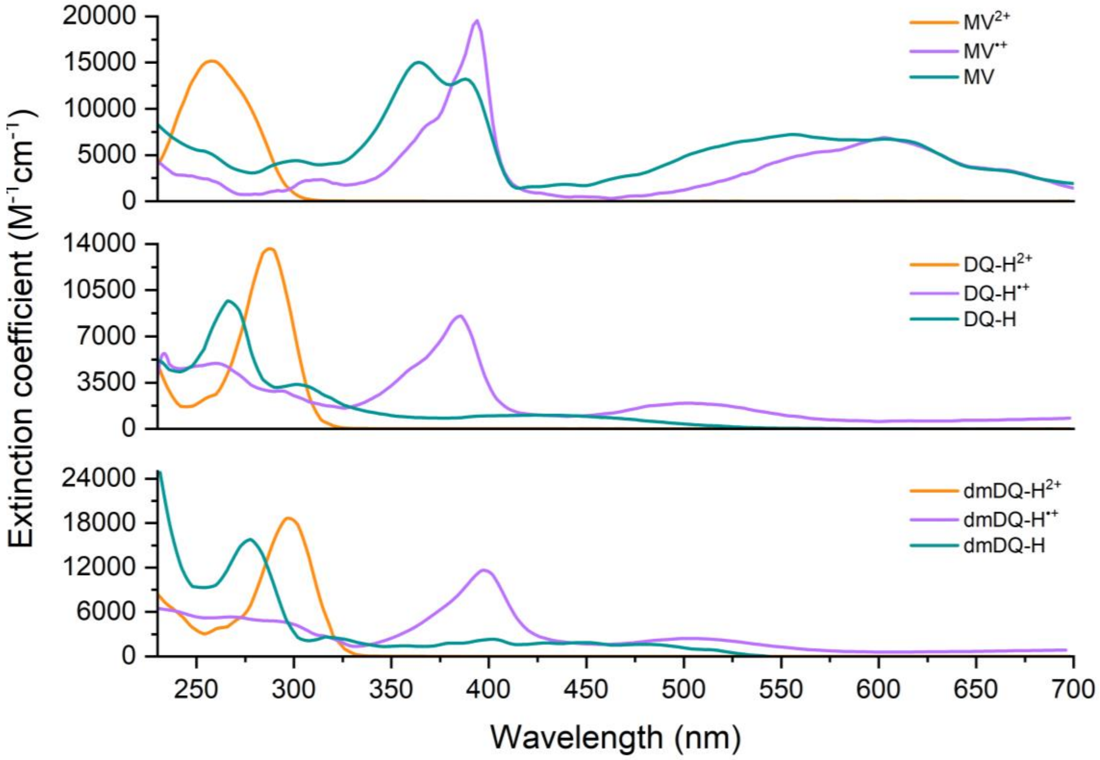

Figure S20. Spectra from deconvolution. Spectra of each non-reduced (experimental data), one-electron reduced and two-electron reduced (deconvolution data) redox mediator in orange, purple and dark cyan, respectively. Spectra of the MV species are shown on top, DQ-H in the middle and dmDQ-H on the bottom together with their calculated extinction coefficients (*y*-axis). The deconvolution simulations showed no contribution of Eu(II) and Eu(III) to the measured spectra and they are therefore not shown in the figure.

H_2 calibration curves

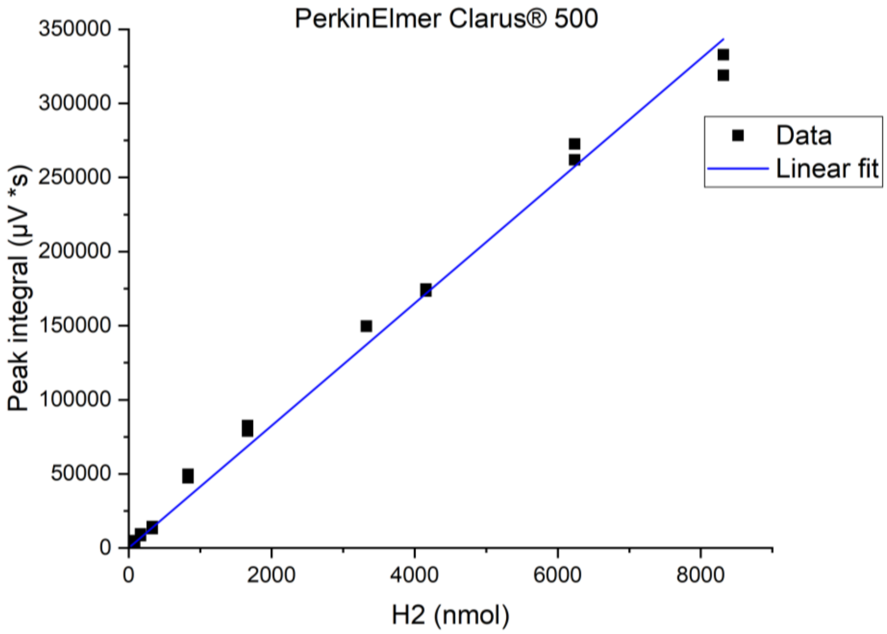

Figure S21. H_2 calibration curve PerkinElmer Clarus® 500. Calibration curve used to convert measured H_2 peak integrals to quantities using gas chromatography. The linear fit yields the equation $y = 41.29337*x$, where y is the H_2 peak integral and x is the amount of H_2 in nmol.

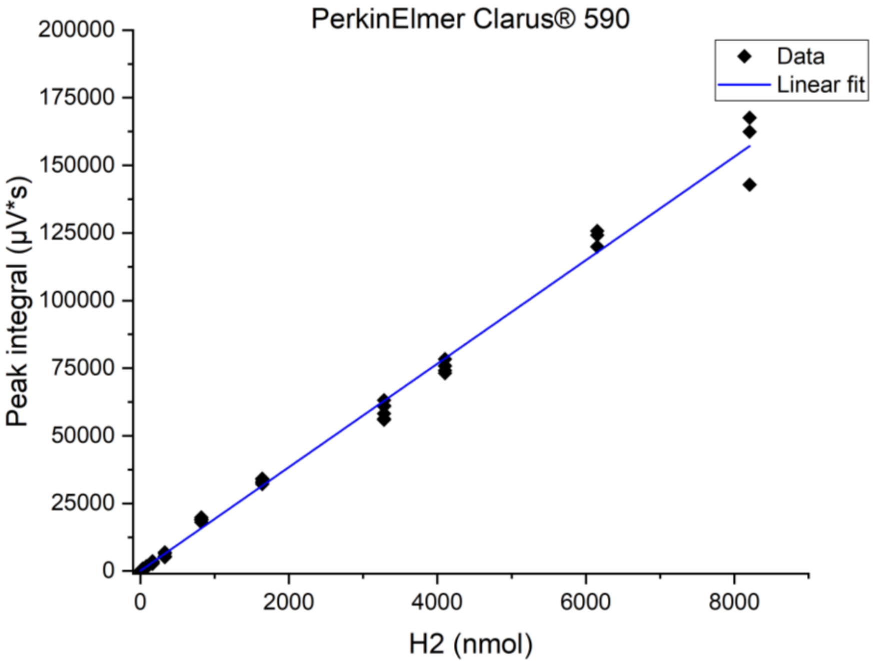

Figure S22. H_2 calibration curve PerkinElmer Clarus®590. Calibration curve used to convert measured H_2 peak integrals to quantities using gas chromatography for Figure 4. The linear fit yields the equation y = 19.13947*x, where y is the H_2 peak integral and x is the amount of H_2 in nmol.

Spectrum of Ti(III)citrate

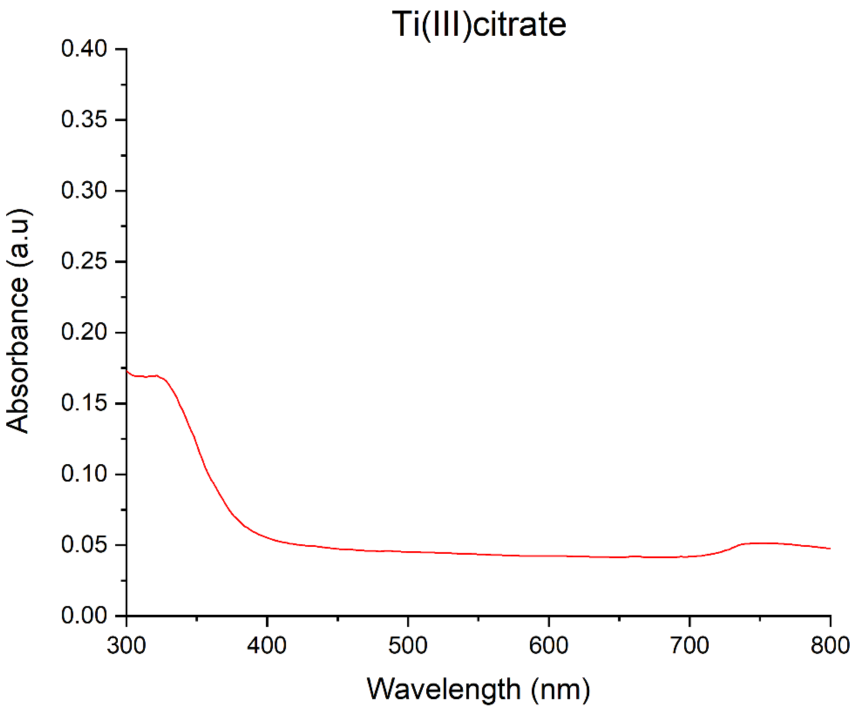

Figure S23. Spectrum of synthesized Ti(III)citrate. The spectrum was found to be in agreement with previously published spectra for Ti(III)citrate. The shape at 340 nm indicates that a small proportion of the sample is in its oxidized form, Ti(IV)citrate. The concentration of Ti(III)citrate was determined using its previously reported extinction coefficient at 340 nm of 0.73 $mM^{-1}cm^{-1}$.[4]

Supplementary notes

Supplementary note 1. Extended discussion on Marcus theory analysis

The rate enhancements observed in solution assays – such as the 35-fold increase from MV to dmDQ-H at pH 8 – significantly exceed those predicted by Marcus theory under standard assumptions. According to Marcus theory, the rate of electron transfer (k_{et}) depends on the reorganization energy (λ), electronic coupling between donor and acceptor (H_{AB}), and the driving force (ΔG).

$$k_{et} = \frac{2\pi}{\hbar}|H_{AB}|^2 \frac{1}{\sqrt{4\pi\lambda k_B T}} \exp\left(-\frac{(\lambda + \Delta G^{\circ})^2}{4\lambda k_B T}\right)$$

By definition, Marcus theory models electron transfer between a single donor and acceptor. In our system, the mediator likely interacts with the surface-exposed FeS-cluster, although literature does not conclusively identify this site. In hydrogenase studies, the driving force is typically calculated relative to the catalytic reaction, assuming fast intramolecular steps.[9, 10]

With this latter assumption, ΔG is estimated from the difference between the solution potential (set by mediator) and the thermodynamic potential of the H^+/H_2 couple. Assuming that H_{AB} remains constant across different mediators, we can eliminate this term by comparing rate constant at varying driving forces.

$$\frac{\boldsymbol{k_{et\,(1)}}}{\boldsymbol{k_{et\,(2)}}} = \sqrt{\frac{\boldsymbol{\lambda_{(2)}}}{\boldsymbol{\lambda_{(1)}}}}\,\mathbf{exp}\left(-\frac{\left(\boldsymbol{\lambda_{(1)}} + \Delta\mathbf{G}^{\circ}_{(1)}\right)^{2}}{4\boldsymbol{\lambda_{(1)}k_{B}T}} + \frac{\left(\boldsymbol{\lambda_{(2)}} + \Delta\mathbf{G}^{\circ}_{(2)}\right)^{2}}{4\boldsymbol{\lambda_{(2)}k_{B}T}}\right)$$

The total reorganization energy comprises contributions from both the protein and the mediator. The protein contribution is assumed constant if the electron transfer mechanism remains unchanged. Thus, we approximate $\lambda_{(1)} \approx \lambda_{(2)}$.

Experimentally, we observe a substantial increase in maximum reaction rates at pH 8 when switching from MV (500 s^{-1}) to DQ-H (3500 s^{-1}) and dmDQ-H (16500 s^{-1}), corresponding to ~90 mV and ~180 mV increases in driving force. To account for these rate enhancements using Marcus theory, reorganization energies of ~0.5 eV would be required. However, DFT-calculated reorganization energies for FeS-clusters and the H-cluster in [FeFe]-hydrogenases are significantly higher (≥1.7 eV)[11], and mediator reorganization energies are ~0.54-0.68 eV[12]. This suggests a total system reorganization energy of ≥2.24 eV, which is at least four times higher than the value predicted from the Marcus theory-based analysis. An estimate using a more realistic λ of 2.3 eV predicts rate increases from 500 s^{-1} (MV) to 2810 s^{-1} (DQ-H) and 3405 s^{-1} (dmDQ-H), which are far below the observed values.

Alternatively, we can calculate ΔG assuming electron transfer to the surface-exposed FeS-cluster. Reported redox potentials for this cluster vary: Artz et al. report ≤−450 mV vs SHE[8], while Lubner et al. report −565 mV vs NHE at pH 8.8[13]. Further, the pH dependence of the F(or relay)-cluster potential differ between reports.[13, 14] Our data show pH-dependent catalytic turnover, suggesting that the driving force (and thus the potential of the acceptor site) is pH-dependent, as changes in coupling or λ with pH are unlikely. The reorganization energy of the surface exposed FeS-cluster is not directly reported, but inner-sphere λ for [4Fe-4S] clusters is ~0.64 eV.[15] Electron transfer between clusters has a total λ of ~2.2 eV, with significant outer-sphere contributions.[11] Even under the unrealistic assumption of equal contributions from adjacent clusters, the minimum λ for the surface-exposed cluster is ~1.1 eV. Combined with the mediator λ, the total system λ is ~1.6-1.8 eV.

Assuming a cluster potential of −565 mV, electron transfer from MV and DQ-H to the surface exposed cluster would be uphill, and the observed rate increases would imply negative λ values which is not realistic. Assuming −450 mV vs SHE (only ~20 mV different from the potential of the catalytic reaction) yields similar λ estimates (~0.5 eV), still inconsistent with known values.

In summary, regardless of the assumed acceptor site, the observed rate enhancements cannot be fully explained by Marcus theory alone. This supports our conclusion in the main text that the measured rate increases significantly exceed those predicted from Marcus theory, even under different assumptions.

References

(1) Senger, M.; Kernmayr, T.; Lorenzi, M.; Redman, H. J.; Berggren, G. Hydride state accumulation in native [FeFe]-hydrogenase with the physiological reductant H_2 supports its catalytic relevance. *Chem Commun* **2022**, *58* (51), 7184-7187. DOI: 10.1039/d2cc00671e.
(2) Senger, M.; Duan, J.; Pavliuk, M. V.; Apfel, U. P.; Haumann, M.; Stripp, S. T. Trapping an Oxidized and Protonated Intermediate of the [FeFe]-Hydrogenase Cofactor under Mildly Reducing Conditions. *Inorg Chem* **2022**, *61* (26), 10036-10042. DOI: 10.1021/acs.inorgchem.2c00954.
(3) Collins, J. M.; Uppal, R.; Incarvito, C. D.; Valentine, A. M. Titanium(IV) citrate speciation and structure under environmentally and biologically relevant conditions. *Inorg Chem* **2005**, *44* (10), 3431-3440. DOI: 10.1021/ic048158y.
(4) Seefeldt, L. C.; Ensign, S. A. A Continuous, Spectrophotometric Activity Assay for Nitrogenase Using the Reductant Titanium(III) Citrate. *Anal Biochem* **1994**, *221* (2), 379-386. DOI: 10.1006/abio.1994.1429.
(5) Zehnder, A. J.; Wuhrmann, K. Titanium (III) citrate as a nontoxic oxidation-reduction buffering system for the culture of obligate anaerobes. *Science* **1976**, *194* (4270), 1165-1166. DOI: 10.1126/science.793008.
(6) Senger, M.; Mebs, S.; Duan, J.; Shulenina, O.; Laun, K.; Kertess, L.; Wittkamp, F.; Apfel, U.-P.; Happe, T.; Winkler, M.; et al. Protonation/reduction dynamics at the [4Fe–4S] cluster of the hydrogen-forming cofactor in [FeFe]-hydrogenases. *Phys Chem Chem Phys* **2018**, *20* (5), 3128-3140, 10.1039/C7CP04757F. DOI: 10.1039/C7CP04757F.
(7) Martini, M. A.; Rüdiger, O.; Breuer, N.; Nöring, B.; DeBeer, S.; Rodríguez-Maciá, P.; Birrell, J. A. The Nonphysiological Reductant Sodium Dithionite and [FeFe] Hydrogenase: Influence on the Enzyme Mechanism. *J Am Chem Soc* **2021**, *143* (43), 18159-18171. DOI: 10.1021/jacs.1c07322.
(8) Artz, J. H.; Mulder, D. W.; Ratzloff, M. W.; Lubner, C. E.; Zadvornyy, O. A.; LeVan, A. X.; Williams, S. G.; Adams, M. W. W.; Jones, A. K.; King, P. W.; et al. Reduction Potentials of [FeFe]-Hydrogenase Accessory Iron-Sulfur Clusters Provide Insights into the Energetics of Proton Reduction Catalysis. *J Am Chem Soc* **2017**, *139* (28), 9544-9550. DOI: 10.1021/jacs.7b02099.
(9) Armstrong, F. A.; Evans, R. M.; Hexter, S. V.; Murphy, B. J.; Roessler, M. M.; Wulff, P. Guiding Principles of Hydrogenase Catalysis Instigated and Clarified by Protein Film Electrochemistry. *Acc Chem Res* **2016**, *49* (5), 884-892. DOI: 10.1021/acs.accounts.6b00027.
(10) Fasano, A.; Land, H.; Fourmond, V.; Berggren, G.; Léger, C. Reversible or Irreversible Catalysis of H^+/H_2 Conversion by FeFe Hydrogenases. *J Am Chem Soc* **2021**, *143* (48), 20320-20325. DOI: 10.1021/jacs.1c09554.
(11) McCullagh, M.; Voth, G. A. Unraveling the role of the protein environment for [FeFe]-hydrogenase: a new application of coarse-graining. *J Phys Chem B* **2013**, *117* (15), 4062-4071. DOI: 10.1021/jp402441s.
(12) Lv, Y.; Liu, Y.; Feng, T.; Zhang, J.; Lu, S.; Wang, H.; Xiang, Y. Structure reorganization-controlled electron transfer of bipyridine derivatives as organic redox couples. *J Mater Chem A* **2019**, *7* (47), 27016-27022. DOI: 10.1039/c9ta08910a.
(13) Lubner, C. E.; Artz, J. H.; Mulder, D. W.; Oza, A.; Ward, R. J.; Williams, S. G.; Jones, A. K.; Peters, J. W.; Smalyukh, I. I.; Bharadwaj, V. S.; et al. A site-differentiated [4Fe–4S] cluster controls electron transfer reactivity of *Clostridium acetobutylicum* [FeFe]-hydrogenase I. *Chem Sci* **2022**, *13* (16), 4581-4588. DOI: 10.1039/d1sc07120c.
(14) Caserta, G.; Papini, C.; Adamska-Venkatesh, A.; Pecqueur, L.; Sommer, C.; Reijerse, E.; Lubitz, W.; Gauquelin, C.; Meynial-Salles, I.; Pramanik, D.; et al. Engineering an [FeFe]-Hydrogenase: Do Accessory Clusters Influence O_2 Resistance and Catalytic Bias? *J Am Chem Soc* **2018**, *140* (16), 5516-5526. DOI: 10.1021/jacs.8b01689.
(15) Sigfridsson, E.; Olsson, M. H.; Ryde, U. Inner-sphere reorganization energy of iron-sulfur clusters studied with theoretical methods. *Inorg Chem* **2001**, *40* (11), 2509-2519. DOI: 10.1021/ic000752u.
